# Phylogeography of the *Anaspides richardsoni* species clade (Anaspidacea, Anaspidesidae): glaciation and recolonization of the Tasmanian Central Plateau and the question of paraphyletic species

**DOI:** 10.1111/cla.70005

**Published:** 2025-07-28

**Authors:** Christoph G. Höpel, Shane T. Ahyong, Martin Kapun, Martin Schwentner, Stefan Richter

**Affiliations:** ^1^ Allgemeine & Spezielle Zoologie, Institut für Biowissenschaften Universität Rostock Universitätsplatz 2 18055 Rostock Germany; ^2^ Australian Museum Research Institute Australian Museum 1 William Street Sydney NSW 2010 Australia; ^3^ School of Biological, Earth & Environmental Sciences University of New South Wales Kensington NSW 2052 Australia; ^4^ Naturhistorisches Museum Wien Burgring 7 1010 Wien Austria

## Abstract

We herein present a phylogenetic and population genetic analysis of a Tasmanian Mountain Shrimp clade, based on ddRAD and cytochrome oxidase subunit‐1 data sets. Our data show that the morphologically well‐delineated and widespread *Anaspides richardsoni* Ahyong, 2016 is paraphyletic with respect to four other species (*A. eberhardi* Ahyong, 2016, *A. spinulae* Williams, 1965 and two undescribed species). These four species all form discrete (monophyletic) lineages and exhibit clear morphological distinctions in relation to *A. richardsoni* and to one another. However, we detect signals of introgression between some populations of *A. richardsoni*, *A. spinulae* and an undescribed species. We also find two instances of syntopic occurrences without evidence for interbreeding. Also, *A. richardsoni* is split into several allopatric and comparably old lineages. *Anaspides spinulae* from Lake St. Clair, however, seems to be a young species that might have differentiated only after the last glacial maximum of central Tasmania (22 000–17 000 years ago). Moreover, we analyse the present population structure and recolonization of the Central Plateau and Western Mountain Ranges in regard to their glacial history. We distinguish several glacial refugia and show that the recolonization most likely occurred only from one or two of these.

## Introduction

The mountain shrimps of the genus *Anaspides* are endemic to Tasmania and represent one of the most “iconic” freshwater invertebrates of the island. Since their discovery more than a century ago (Thomson, 1893), they have attracted significant scientific interest as a kind of “living fossil” (closely resembling their Triassic ancestors/relatives; Brooks, 1962). For most of the time since its discovery, *Anaspides* was assumed to include only a single species, *A. tasmaniae* (Thomson, 1893), that was widespread over most parts of Tasmania in various freshwater bodies such as streams, tarns, lagoons, lakes and caves. Based on distinct but variable morphological characters and being apparently reproductively isolated from nearby populations of *A. tasmaniae*, a second species, named *A. spinulae* Williams, 1965, was described only in 1965 from Lake St. Clair at the edges of the Central Plateau.

**Fig. 1:**
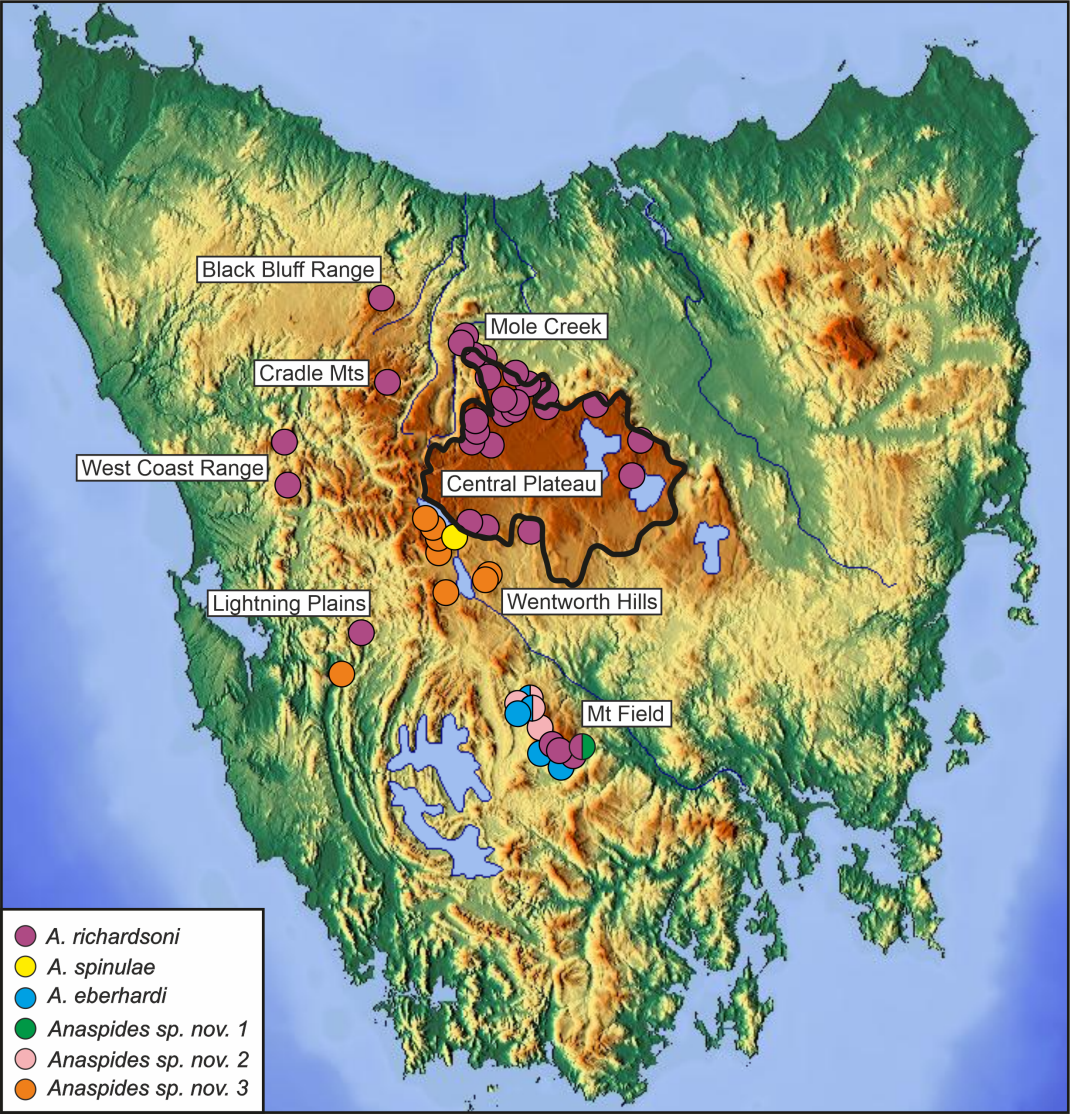
Topographical map of Tasmania with collection sites of the six species of *Anaspides* (forming the “central clade”) examined in this study. Central Plateau marked with a black line (after Corbett, 2019). Purple: *A. richardsoni*, Yellow: *A. spinulae*, Blue: *A. eberhardi*, Green: *Anaspides* sp. nov. 1 from Lake Nicholls, Pink: *Anaspides* sp. nov. 2 from the Florentine Valley, Orange: *Anaspides* sp. nov. 3. Halved circles indicate syntopic occurrence of different species. Modified after the topographic map from Wikipedia (https://upload.wikimedia.org/wikipedia/commons/2/21/Topography_of_Tasmania.jpg, common licence under Creative Commons Attribution‐ShareAlike 3.0 Unported).

To date, eight species of *Anaspides* are recognized (Ahyong, 2015, 2016; Höpel et al., 2023a), of which two are obligate cave dwellers (*A. clarkei* Ahyong, 2015, *A. eberhardi* Ahyong, 2016) and three have surface as well as cave populations (*A. jarmani* Ahyong, 2015, *A. richardsoni* Ahyong, 2016, *A. swaini* Ahyong, 2015). The three remaining species (*A. driesseni* Höpel et al., 2023a, *A. spinulae*, *A. tasmaniae*) are only known from surface habitats. According to Richter et al. (2018) and Höpel et al. (2023b), the widespread *A. richardsoni* and the more narrowly distributed species, *A. eberhardi* (Junee‐Florentine karst at Mt. Field) and *A. spinulae* (Lake St. Clair), form a species group, therein called the “central clade,” alluding to its central Tasmanian distribution (Fig. 1). The distribution of the “central clade” includes the Central Plateau, a highland area mostly above 1000 m with ridges that steeply drop 500–700 m to the west and north, which slopes more gently to the south (Corbett, 2019), but also the Western Mountain Ranges (e.g. West Coast Range, Cradle Mountains, Black Bluff Range) as well as Mt. Field plateau in central Tasmania. The Central Plateau and the Mt. Field plateau are composed of Jurassic dolorite as the uppermost layer.

Phylogenetic analyses based on data from the mitochondrial (mt) cytochrome oxidase subunit‐1 (COI) marker (Richter et al., 2018) and from mt genomes (Höpel et al., 2023b) indicate that *A. richardsoni* is paraphyletic with *A. eberhardi* and *A. spinulae* nested within. Höpel et al. (2023b) also showed that the species identified by Ahyong (2016) as “*A. swaini*, form 2” belongs to the central clade as well (herein *Anaspides* sp. nov. 3). The molecular clock analysis based on COI data in Richter et al. (2018) suggested that the initial split in the central clade occurred 4 Mya, but that most subsequent splits are younger and might have indeed appeared during the Pleistocene. Because most surface species of *Anaspides* live at high altitudes, the distribution of extant *Anaspides* in Tasmania has been heavily influenced by glaciation during the Pleistocene (e.g. Kiernan, 1990; Kiernan et al., 2001). For example, the previously glaciated parts of the Central Plateau and Mt. Field were re‐colonized after glaciation.

During the Early Pleistocene, vast areas of Tasmania were covered with thick glaciers (Colhoun et al., 2010). The largest were present during the multi‐phased Linda Glaciations, which covered huge areas of the Central Plateau and spanned most of the western mountain regions (Fig. 11). A total area of approximately 7000 km^2^ was covered with a maximum ice thickness of 500–800 m. The outlet glaciers in the valleys still had a thickness of 150–350 m. However, the exact age of this glacial maximum is uncertain. Because of reversed magnetic polarity in varved clays in lake deposits, these glaciations are older than 0.785 Mya (Barbetti and Colhoun, 1988) and, with little doubt, some of these ice advances are up to 2.5 million years old. There is even evidence for glaciations between the Oligocene and Pliocene, which is consistent with glaciations in Patagonia and New Zealand; however, without any dating (Macphail et al., 1993; Augustinus and Idnurm, 1993). The mid‐Pleistocene glaciations (at least three) were less extensive (Colhoun et al., 2010). The last glacial maximum (LGM) occurred from 22 000 to 7000 years ago during marine isotope stage 2. There is again evidence for a couple of succeeding glaciations (e.g. Mackintosh et al., 2006). The LGM glaciations had an extent of 1100 km^2^, a thickness of 250 m and covered the Central Plateau, the Western Mountain Ranges and several mountain ranges in the south and south‐west of Tasmania (Fig. 11).

### Species concepts and phylogeography

The dominant species concept at present is the general lineage species concept (De Queiroz, 1998), where species correspond to segments of population lineages with (definitely) independent trajectories (Vences et al., 2024). The other species “concepts” are suggested as operational “criteria” that emerge gradually during speciation (De Queiroz, 1998, 2007). During speciation, there is a transition from a single population lineage with reticulating relationships among individuals towards bifurcating relationships between two population lineages with progressively reduced gene flow and finally reproductive isolation between these lineages (De Queiroz, 1998). Full reproductive isolation corresponds to the Biological Species Concept (BSC; Mayr, 1942) or to the Hennigian Species Concept (HSC; Meier and Willmann, 2000). In general, de Queiroz’ concept is quite close to Hennig's (1966) concept on speciation as “dissolving tokogenetic relationships” *within* species and the origin of phylogenetic relationships *between* species (and higher taxa). Delimiting lineages into species has often relied on quantifying their genetic divergences, for example, by sequencing the “barcoding gene” COI (Hebert et al., 2003). It is now well known, however, that this is often misleading when hybridization and/or ancestral polymorphism result in mitonuclear discordance (Bonnet et al., 2017). High‐throughput sequencing now allows combining thousands of markers from both the mitochondrial and the nuclear genomes, which obviously allows a much more nuanced analysis of divergences between lineages than the use of a few (or only a single) barcoding genes (Vences et al., 2024).

One important aspect of species delimitation is the integration of biogeographical criteria. If two lineages occur in sympatry without genetic admixture, they conclusively fulfil the biological species criterion (Vences et al., 2022, 2024). Much more challenging are allopatric lineages, where genetic differentiation inferred from SNPs might reflect old phylogeographical structure driven by allopatric evolution (Vences et al., 2024). Dufresnes et al. (2024) suggested a workflow to delimit “phylogeographic lineages” into species and subspecies.

The question, however, is if species must be monophyletic or whether species might also be paraphyletic (“paraspecies” *sensu* Ackery and Vane‐Wright, 1984) (Crisp and Chandler, 1996). In a strict sense, Hennig (1966) applied the term monophyletic and paraphyletic only for taxa above the species‐level because within the conceptual framework of Hennig's argumentation the stem species becomes extinct during the process of speciation, which formally excludes the possibility of paraphyletic species (see also Meier and Willmann, 2000). However, if the focus is placed on the speciation process itself (instead of its formalized outcome), it is quite obvious that if new species arise by a gradual process (e.g. by evolving ecological differentiation and isolating mechanisms) from an allopatric population within a wide‐spread species, the “remainders” of the original species would not necessarily evolve in the same timeframe and manner. These “remainders” then might still consist of various (allopatric) populations (Barraclough, 2019) as, for example, found for the island endemic *Aphelocoma insularis*, which arose from the Western scrub‐jay (*Aphelocoma californica*), a widely distributed mainland bird species in the USA (Delayne, 2014). This would most probably result in what could be considered a paraphyletic species (if no isolating mechanisms arise between these “remaining” populations). Interestingly, this might not only be the outcome of allopatric speciation but also of sympatric speciation (e.g. Barluenga et al., 2006), which is in good concordance with the so‐called Phylogenetic Species Concept (PSC), where species are “the smallest diagnosable cluster of self‐perpetuating organisms that have unique sets of [morphological] characters” (Nelson and Platnick, 1981; see also Nixon and Wheeler, 1990, 1992). This includes the possibility that a species might be characterized only by plesiomorphic characters which would be expected for paraphyletic species.

Coyne and Orr (2004) in their book on “Speciation” discuss the problem of paraphyletic species in the light of the Biological Species Concept (which in our view can be adopted to the General Lineage Concept, GLC). They suggest that most cases of reported “paraphyletic species” are an analytical artefact of the use of only a few (mostly) mitochondrial genes. The main problem they describe is the supposed ongoing gene flow between the population of the “paraphyletic species” which might not be correctly discovered by analyses using only a few genes. They recognize, however, that “it is likely that some multi‐gene phylogenies may show biological species to be truly paraphyletic, and that the relatedness of populations and individuals may not always be concordant with their assignment to biological species” (Coyne and Orr, 2004).

Herein, we present a new analysis of the “central clade” *Anaspides* using ddRAD data as well as COI. The ddRAD data allow a remarkable population‐level resolution. Populations are first characterized by the collecting locality that, in the case of the surface populations, are (mostly) (micro)allopatric water bodies such as tarns and lakes. We discuss the geographic separation of these habitats in correlation with the genetic isolation of the inhabitants (a case of “reciprocal illumination”).

This study has two major aims. Based on the relationships of the populations, we aim for a causal understanding of species differentiation and speciation in terms of allopatric lineages and the acquisition of new morphological characters. The second aim is to correlate population differentiation with paleoecological events, namely glaciation, in order to develop our causal understanding of the observed pattern.

## Materials and methods

Specimens were identified using the morphological descriptions of Ahyong (2015, 2016). The three as yet undescribed species can also be diagnosed by morphological characters used by Ahyong (2015, 2016) and Höpel et al. (2023a).

Specimens for this study were collected during five field trips in 2017, 2019, 2022, 2023 and 2024. A total of 347 specimens from 48 localities were examined and newly sequenced for this study (Table S1). All sequences were deposited at GenBank (Benson et al., 2003) (COI: PV780789–PV781115; ddRAD: SAMN49064136–SAMN49064398, BioProject ID: PRJNA1276447).

The DNA extraction was performed with the DNeasy Blood and Tissue Kit (Qiagen, Hilden, Germany), following the manufacturer's instructions. Mitochondrial cytochrome c oxidase subunit I (COI) was partially sequenced. The polymerase chain reaction (PCR) had a total volume of 30 μL containing 3 μL of each primer, 3 μL 10× PCR buffer (Sigma‐Aldrich, St. Louis, MO, USA), 3 μL dNTP mix (Biozym, Hessisch Oldendorf, Germany), 2.58 μL MgCl_2_ (Sigma‐Aldrich), 0.12 μL Taq‐polymerase (Sigma‐Aldrich), 10.71 μL ultrapure water, and 4.5 μL of the DNA extract. Primers were LCO2 (50‐TCN ACH AAY CAT AAA GAY ATT GGA AC‐30) (Richter et al., 2018), HCO2198 (50‐TAA ACT TCA GGG TGA CCA AAA AAT CA‐30) (Folmer et al., 1994). PCR amplification programs comprised an initial denaturation step at 94 °C for 1 min, followed by 40 amplification cycles (94 °C for 30 s, 50 °C for 30 s, 72 °C for 60 s) and a final elongation step at 72 °C for 5 min. All PCR reactions were performed with a Mastercycler gradient (Eppendorf, Hamburg, Germany). PCR products were visualized by gel electrophoresis, using 5 μL of the PCR product with 1.5 μL loading buffer DNA b II (AppliChem) on a 1.2% agarose/TAE gel stained with 5 μL Rotisafe (Carl Roth, Karlsruhe, Deutschland). PCR products were purified using paramagnetic beads (High Prep PCR; Magbio, Lausanne, Schweiz) following the manufacturer's instructions with a final volume of 25 μL. Sequencing of PCR products was performed in Lightrun96 well plates by the company Eurofins Genomics. The resulting chromatograms were manually checked and adjusted with Geneious 2019.1.3 (Biomatters Limited, Auckland, New Zealand). The sequences of the COI gene fragment were aligned using GeneiousAlign. Haplotype networks were calculated with Network 5.0.1.1 (Fluxus Technologies, Stanway, UK) by using the median‐joining method. A maximum likelihood phylogeny was calculated using the RaxML GUI (Darriba et al., 2020) with the best model (HKY + G + I) determined by the implemented model test. The calculation was run using the thorough bootstrap analysis for 1000 bootstraps.

Molecular clock analyses were performed in BEAST 2.4.6 (Bouckaert et al., 2014). The input files for BEAST were created with Beauti as part of the BEAST package. Mutation rates for COI were based on averages of reported mutation rates for Crustacea: 1.4% per million years (Knowlton and Weigt, 1998), 1.66% per million years (Schubart et al., 1998) and 2.33% per million years (Schubart et al., 1998) for COI (average per lineage 0.9% per million years). In addition to setting the mean rates, the original rates were set as maximum or minimum rate constraints, respectively. A strict clock with a Yule tree prior with standard parameters was employed as well as the GTR + I + G nucleotide substitution model. BEAST was run for 110 mio generations, saving every 10 000th tree. Stable likelihood plateaus and convergence were assessed with Tracer v1.7.1 (Rambaut et al., 2018). The first 20% of trees were discarded as burn‐in, and the posterior probability limit was set to 0.5 in TreeAnnotator 2.4.6 (Bouckaert et al., 2014).

### ddRAD data analyses

For ddRAd analyses, we largely followed the protocol by Schwentner and Lörz (2021). For example, we added four random nucleotides in one adapter to allow the downstream detection and removal of PCR duplicates (similar to Franchini et al., 2017). The DNA concentration of each DNA extract was quantified on a Qubit 3.0 (Invitrogen). Of each DNA extract, 150–250 ng DNA was diluted in 24 μL with H_2_O. Samples were always processed in batches of eight, which were pooled prior to PCR amplification (all of these eight share the index added by PCR but received individual barcodes during adapter ligation; Table S1) and which always had the same DNA concentration. 1.5 μL fastdigest MspI, 1.5 μL fastdigest EcoRI and 3 μL 10× buffer (all Thermo Fisher) were added to each DNA sample and incubated for at least 4 h at 37 °C. Digested DNA was cleaned with 45 μL magnetic beads (AmpliClean Cleanup Kit, Nimagen) following the manufacturer's recommendations and eluted with 21 μL H_2_O, of which 19 μL were transferred for adapter ligation. The above‐mentioned steps were repeated for a few samples as internal technical replicates to ascertain reproducibility of the results. These samples were digested twice and each of these replicates was treated like an independent sample (e.g. receiving its own barcode/index combination, etc.). All adapters were diluted in annealing buffer (10× annealing buffer comprises 500 mM NaCl, 100 mM Tris‐Cl, 10 mM EDTA) to 2 μM (EcoRI adapters) or 31.6 μL (MspI adapters), respectively (following Peterson et al., 2012). Sixteen EcoRI and 8 MspI adapter variations with unique barcodes (MspI adapters with additionally four random nucleotides following the barcode: Franchini et al., 2017) were used to tag each library with a unique adapter combination within each batch of eight samples. Barcode sequences were derived from Peterson et al. (2012). Ligation reactions comprised 19 μL digested DNA, 3 μL EcoRI adapter, 3 μL MspI adapter, 2 μL T4 Ligase (2 Weiss units; Thermo Fisher Scientific, Waltham, MA, USA) and 3 μL 10× T4 ligase buffer and was incubated at 22 °C for 1 h and then heat killed at 65 °C for 10 min. Subsequently, the eight samples of each batch were pooled and each pool cleaned with 360 μL magnetic beads, eluting in 32 μL TE buffer. Size selection was carried out on a BluePippin (1.5% dye free cassettes with marker L; Biozym) for each pool independently, selecting the “tight” option with a mean size of 300 bp (range 261–339 bp). To reduce amplification biases during PCR, four PCR replicates were run for each pool. Each PCR comprised 0.3 μL (10 nM) of each primer, 5 μL 2× Kappa HIFI HotStart ReadyMix (Roche, Basel, Switzerland) and 4.4 μL of a pool after size selection. A two‐step PCR program of 95 °C initial denaturation and 12–15 cycles of 98 °C for 20 s and 72 °C for 25 s plus a final elongation step of 1 min at 72 °C was run. Forward and reverse primers were available with eight different 8 bp indices each and combined to add a unique index combination for each pool (Table S1). After PCR, the four PCR replicates of each pool were mixed and cleaned with 40 μL magnetic beads (eluting in 20 μL H_2_O). Concentrations of all cleaned pools were measured with Qubit 3.0, and correct fragment size length (~350 bp) was assessed on a Tapestation (D1000 ScreenTape; Agilent, Santa Clara, CA, USA). All libraries were mixed at equal molarity into a final pool of 20 nM in 60 μL. Paired‐end sequencing with 100 bp each was carried out on one Illumina HiSeq4000 lane by Macrogen (Seoul, South Korea). Upon sequencing, Macrogen pre‐demultiplexed the data into the respective pools (based on provided index combinations).

Using STACKS v2.59 (Rochette et al., 2019), PCR duplicates were removed using the clone_filter program, and subsequently, the process_radtags program was used to demultiplex, these pools into the individuals by their unique barcodes. The program denovo_map.pl was then used to create a catalogue of loci (parameters: ‐m 3, ‐M 7, ‐n 7).

**Fig. 2:**
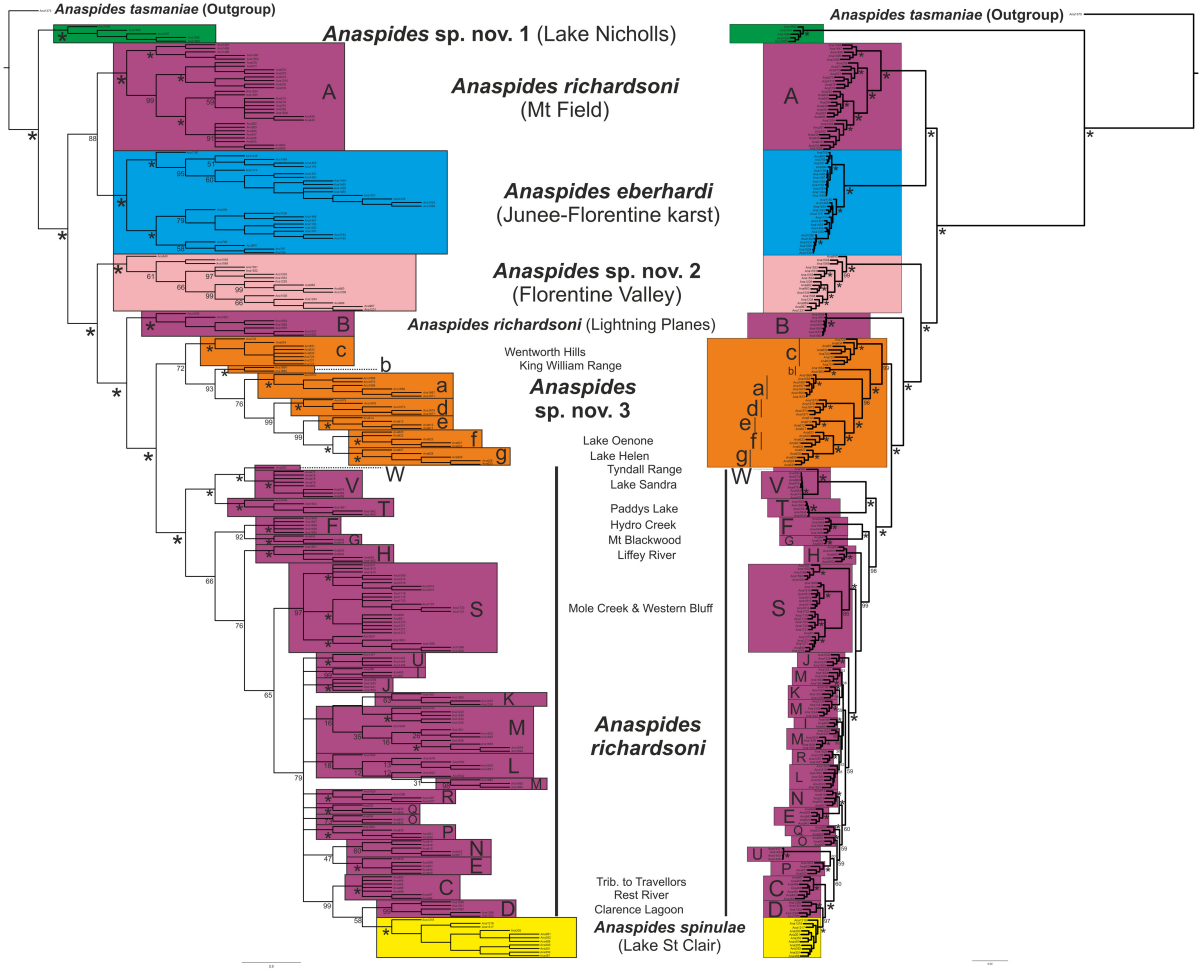
Strict consensus tree of 1000 most parsimonious trees calculated with TNT V1.6 using the script PhylogenomicSupport.run (Torres et al., 2022) (left) and maximum‐likelihood phylogeny calculated with IQ‐Tree 2.3.6 (TVM + G + I, 1000 iterations) of ddRAD data set 1 using *Anaspides tasmaniae* as outgroup (261 individuals, 1945 polymorphic loci with 38 926 variable sites). The letters correspond to the populations/metapopulations in Fig. 11. Support values are only shown for the deep branches and major lineages. Support values for MP analysis were calculated using the PhylogenomicsSupport.run script (Torres et al., 2022). Best score for MP analysis was 48 021. An asterisk (*) indicates support value of 100. Coloured boxes correspond to morphologically delineated species (based on Ahyong, 2015, 2016; Höpel et al., 2023a). Green: *Anaspides* sp. nov. 1, Purple: *A. richardsoni*, Blue: *A. eberhardi*, Light Pink: *Anaspides* sp. nov. 2, Orange: *Anaspides* sp. nov. 3, Yellow: *A. spinulae*. Most localities mentioned in the text are given for the respective clade.

Four data sets were generated using the populations program of STACKS v2.59 (Rochette et al., 2019) with the parameter ‐R set to 0.89 (Table 1). The first and second data sets comprised 261 and 260 specimens, respectively, with data set 1 including one specimen of *A. tasmaniae* which was used as an outgroup in the subsequent tree analyses. Specimens with more than 30% missing loci were excluded from the analyses (except for the outgroup). The third data set comprised 177 specimens of the more derived lineages (based on the previous tree analyses of the first data set, see Fig. 2). Again, specimens with more than 30% missing loci were excluded. In this data set, we could more than double the number of polymorphic loci in comparison to data sets 1 and 2. This allows us to analyse the population structure, admixture and potential introgression within these derived lineages at much higher resolution and detail. It is also necessary to facilitate a detailed analysis of potential glacial refuges and recolonization paths. We also generated a fourth data set including only specimens from Clarence Lagoon, Tributary to Travellers Rest River (both *A. richardsoni*) and Lake St. Clair (*A. spinulae*) to get a fine‐scale resolution of this group.

**Table 1:** Data sets with number of specimens, loci, polymorphic loci and sites analysed

Data set	Specimen	Loci	Polymorphic loci	Variable sites (SNPs)
1	261	1963	1945	38 926
2	260	1981	1963	38 290
3	177	4024	3976	54 396
4	23	6385	5379	24 633

Phylogenetic relationships between the species, populations and lineages were inferred by maximum likelihood (ML) and maximum parsimony (MP) analyses using the .var.phy file generated by the populations program using STACKS as well as an unrooted phylogenetic network calculated using SplitsTree4 v4.19.2 (Huson and Bryant, 2006) with the Neighbor‐Net algorithm and uncorrected *p*‐distances. We used IQ‐TREE 2 (Minh et al., 2020) to infer the ML tree using the TVM + G + I model and 1000 bootstraps. The best‐fit model of evolution was determined by the implemented model test in RaxML GUI 2.0.8 (Darriba et al., 2020). Maximum‐parsimony analyses were conducted with the console version of TNT v1.6, June 2021 (Goloboff and Catalano, 2016) using New Technology (Torres et al., 2022). The script PhylogenomicSearch.run (Torres et al., 2022) was applied with the following arguments specified: search, nt; level, 10; hits, 5; output, newick; outgroup Ana1375. *Anaspides tasmaniae* (Ana1375) was used as outgroup. Resulting trees were visualized with FigTree v.1.4.2 (Rambaut, 2014) and Corel Draw 2020. The Script PhylogenomicSupport.run was used to obtain support values with the following argument specified: reftree, StrictConsensus_tnt.tre; format, tnt; sites, boot; replic, 1000; search, nt; level, 5; output, newick.

To infer population structure, ancestry and admixture we used fineRADstructure (Malinsky et al., 2018), principal component analysis (PCA) following the ipyrad workflow and analyses of population structure and grouping using STRUCTURE 2.3.4 (Pritchard et al., 2000). The fineRADstructure is a high‐resolution method, which was specifically designed to deal with a very large number of populations. It utilizes haplotype linkage information and constructs a coancestry matrix (a summary of nearest neighbour haplotype relationships; Malinsky et al., 2018). The fineRADstructure analyses were run with default parameters (as suggested by the developers) and results were plotted with the fineRADstructure GUI. STRUCTURE and PCA are also using these multi‐locus genotype data to determine distinct populations and can be used to identify and study hybrid zones as well as for identifying migrants and admixed individuals (Pritchard et al., 2000). Both analyses are only using one randomly chosen SNP per locus to minimize the effect of linkage between neighbouring SNPs, which may bias the analyses. For the STRUCTURE analyses, we tested *k* values, that is, the putative number of ancestral groups, from 1 to 30 and conducted 20 replicate runs for each *k* value. Based on these calculations, we selected the optimal number of clusters using the StructureSelector website (Li and Liu, 2018). We only report the first four PC axes of the PCA as the remaining axes cumulatively only explained less than 3.5% of the total genetic variation. To quantify genetic differentiation among the populations we calculated the *F*_ST_ and *d*_xy_ values using the program populations in STACKS v2.59 for all clusters recovered in the fineRADstructure.

To further test for historical gene flow, we calculated *D*‐statistics/ABBA‐BABA test statistics using DSuite (Malinsky et al., 2021) and estimated introgression events using the program TreeMix (Pickrell and Pritchard, 2012). We tested for historical gene flow between the lineages uncovered in the fineRADstructure analyses. DSuite analyses were carried out using the Dtrios method for all possible population trios with *Anaspides* sp. nov. 1 (data set 2) and *Anaspides richardsoni* (B) (data set 3) set as outgroups. The TreeMix analysis was run for *m* values, that is, the number of independent migration events, ranging from 1 to 10 with 10 replicate runs per *m* value. The optimal number of migration events was calculated with the OptM package (Fitak, 2021) in R.

All resulting trees, plots, statistics and figures were post‐processed with Corel Draw 2020. The complete and well‐documented bioinformatic analysis pipeline including custom scripts and the commands for third‐party programs can be found at Github (https://github.com/ch515/Hoepel_paraphyletic_species).

## Results

### Morphological data

Using the taxonomic/diagnostic morphological characters described/delineated by Ahyong (2016) and Höpel et al. (2023a), we can *a priori* distinguish six species (not including *A. tasmaniae,* which is used as outgroup), of which three are formally described (*A. spinulae*, *A. richardsoni* and *A. eberhardi*) and three are as yet undescribed species (*Anaspides* sp. nov. 1, 2, 3). *Anaspides* sp. nov. 3 was identified by Ahyong (2016) as *A. swaini*, form 2.

*Anaspides richardsoni* and *Anaspides* sp. nov. 1 (so far only known from Lake Nicholls on the east side of Mt. Field) are syntopic, as are *A. eberhardi* and *Anaspides* sp. nov. 2, which co‐occur in two caves in the Florentine Valley.

The number and arrangement of antennular clasping spines in adult males is diagnostic in species of *Anaspides* (Ahyong, 2016; Höpel et al., 2023a). *Anaspides richardsoni* and *A. eberhardi* possess only a single antennular clasping spine in adult males, whereas *A. spinulae* and all three undescribed species have two clasping spines. However, in *Anaspides* sp. nov. 1, these two spines are in a dorso‐ventral plane, instead of an anterior–posterior plane as in all other *Anaspides* with more than one clasping spine. For the “central clade” the presence of two clasping spines is the plesiomorphic condition, being present also in *A. tasmaniae* (but also in the more distantly related *A. swaini* and *A. driesseni*). Clasping spines might play a role in reproductive isolation between species (Ahyong, 2016).

*Anaspides* sp. nov. 1 and *A. eberhardi* have pleopodal endopods only on pleonites 1–4, whereas *A. richardsoni*, *A. spinulae*, *Anaspides* sp. nov. 2 and *Anaspides* sp. nov. 3 have pleopodal endopods also on pleonite 5. Both *Anaspides* sp. nov. 1 and *A. spinulae* mature at a smaller size than their congeners (14–15 mm body length in *A. spinulae*). *Anaspides spinulae* is also characterized by having a telson with a spine row of slender, uneven, closely spaced spines, with several longer spines. Moreover, this species shows pronounced spination of the pleural margins (usually starting on pleuron 1) and the tergal margin 5–6, a characteristic also found in *Anaspides* sp. nov. 2 (starting on pleuron 3–4) and rarely in some populations of *A. richardsoni* (e.g. small pool near Little Throne, some specimens from Clarence Lagoon).

### ddRAD data

**Fig. 3:**
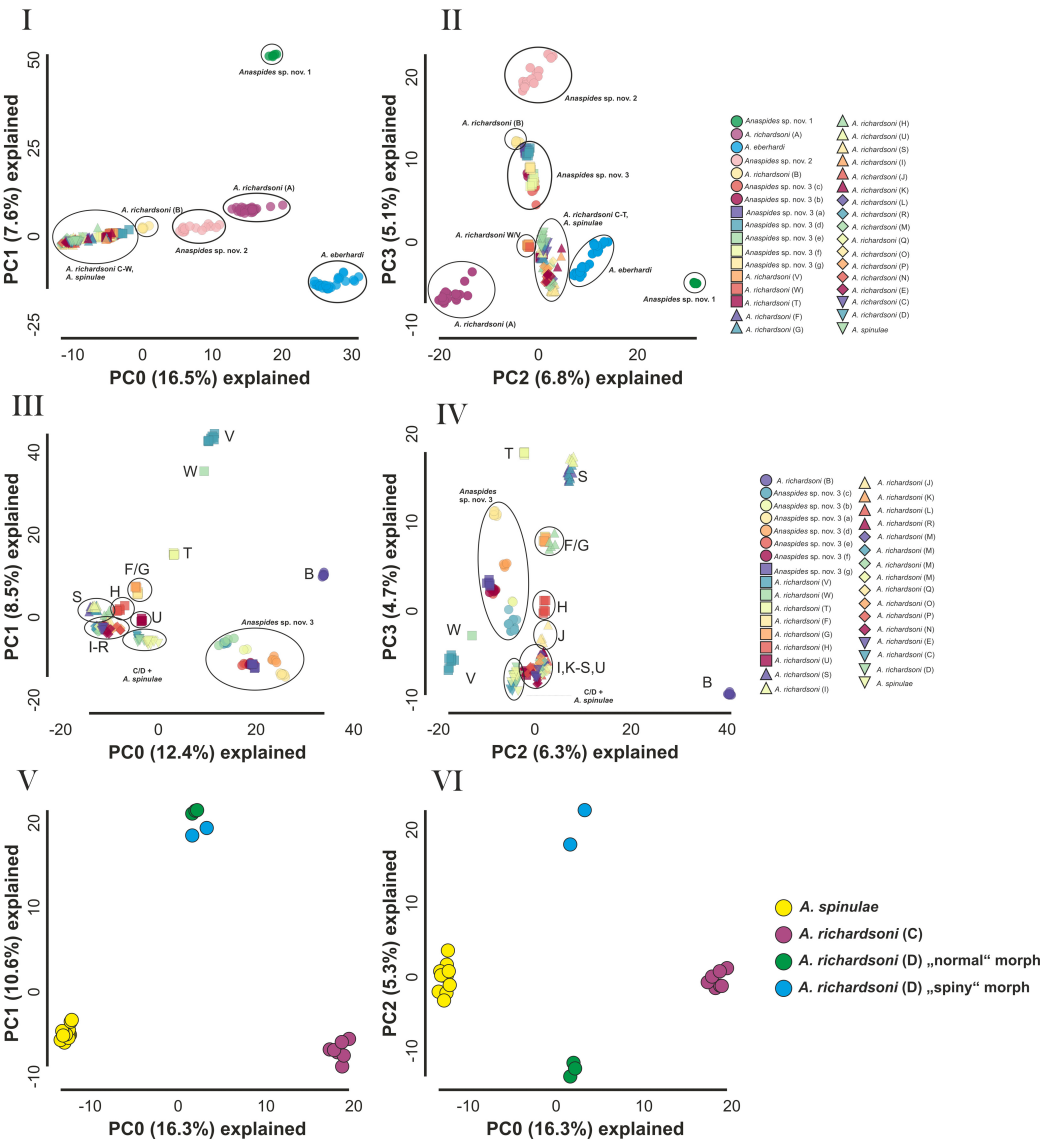
Principal component analyses using data sets 2, 3 and 4. Data set 2: (I) PC0/PC1 and (II) PC2/3; data set 3: (III) PC0/PC1 and (IV) PC2/3; data set 4: (V) PC0/PC1 and (VI) PC0/PC2. The following PCs for data sets 2 and 3 explain less than 3.5% and less than 4.5% for data set 4 and are not shown here. The letters correspond to the populations/metapopulations in Fig. 11.

**Fig. 4:**
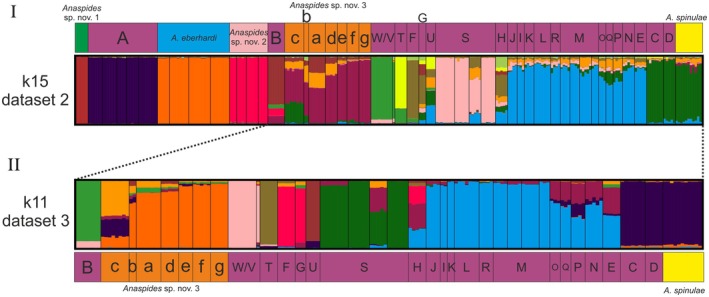
STRUCTURE analysis using data set 2 (I) and data set 3 (II) of the six species analysed in this study. Plots for the best *k* values for each data set are shown (data set 2: k15, data set 3: k11). The letters correspond to the populations/metapopulations in Fig. 11. Coloured boxes correspond to morphologically delineated species (based on Ahyong, 2015, 2016; Höpel et al., 2023a). Green: *Anaspides* sp. nov. 1, Purple: *A. richardsoni*, Blue: *A. eberhardi*, Light Pink: *Anaspides* sp. nov. 2, Orange: *Anaspides* sp. nov. 3, Yellow: *A. spinulae*. Note colours for population structure plots do not match between data sets 2 and 3.

**Fig. 5:**
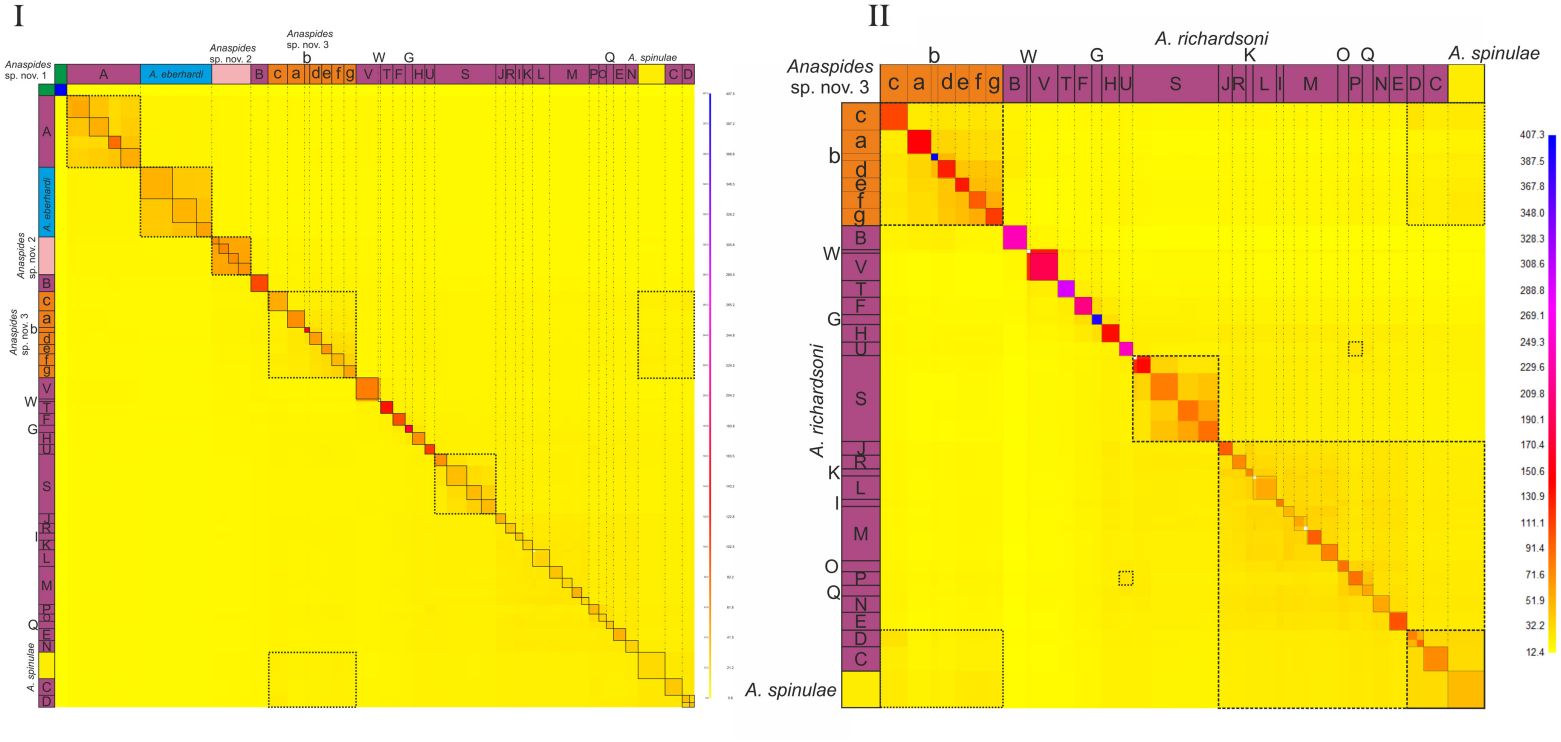
FineRADstructure analyses of the six species analyses in this study for (I) data set 2 and (II) data set 3. Darker colours (range: blue–>yellow) indicate higher shared coancestry. The letters correspond to populations/metapopulations in Fig. 11. Coloured boxes on sides correspond to morphologically delineated species (based on Ahyong, 2015, 2016; Höpel et al., 2023a). Green: *Anaspides* sp. nov. 1, Purple: *A. richardsoni*, Blue: *A. eberhardi*, Light Pink: *Anaspides* sp. nov. 2, Orange: *Anaspides* sp. nov. 3, Yellow: *A. spinulae*. Black closed lined boxes were manually added for better visibility of clusters with higher coancestry representing populations. Dashed broken lines were manually added for better assignment of coloured boxes (letters) with the genetic clusters. Black broken lined boxes were manually added to highlight some areas with increased coancestry.

**Fig. 6:**
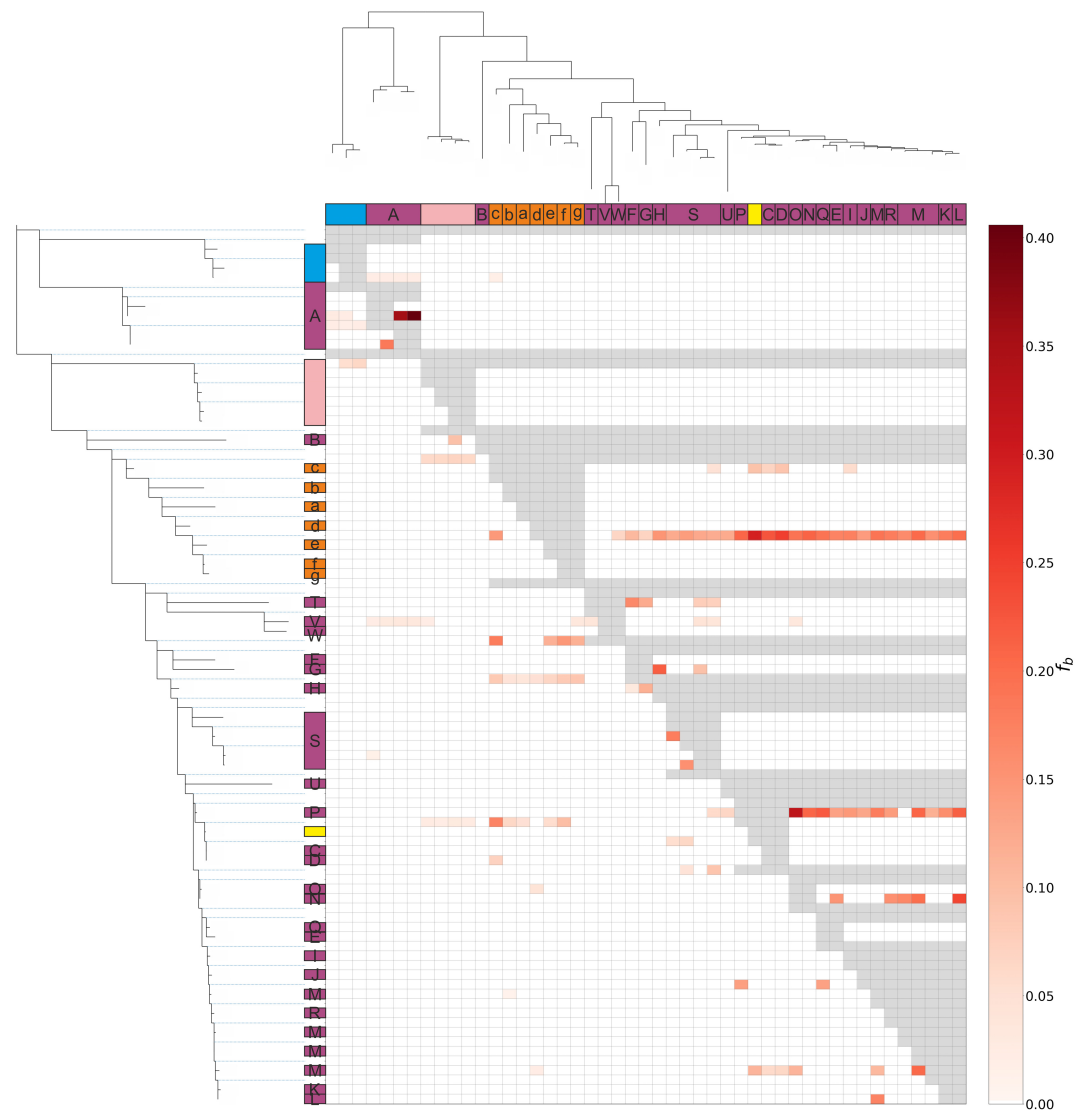
DSuite analysis of ddRAD data set 2 (260 individuals, 1945 polymorphic loci with 38 926 variable sites). *Anaspides* sp. nov. 1 was used as an outgroup. Grey boxes are impossible combinations for Dtrios calculation. The letters correspond to the populations/metapopulations in Fig. 11. Coloured boxes correspond to morphologically delineated species (based on Ahyong, 2015, 2016; Höpel et al., 2023a). Green: *Anaspides* sp. nov. 1, Purple: *A. richardsoni*, Blue: *A. eberhardi*, Light Pink: *Anaspides* sp. nov. 2, Orange: *Anaspides* sp. nov. 3.

**Fig. 7:**
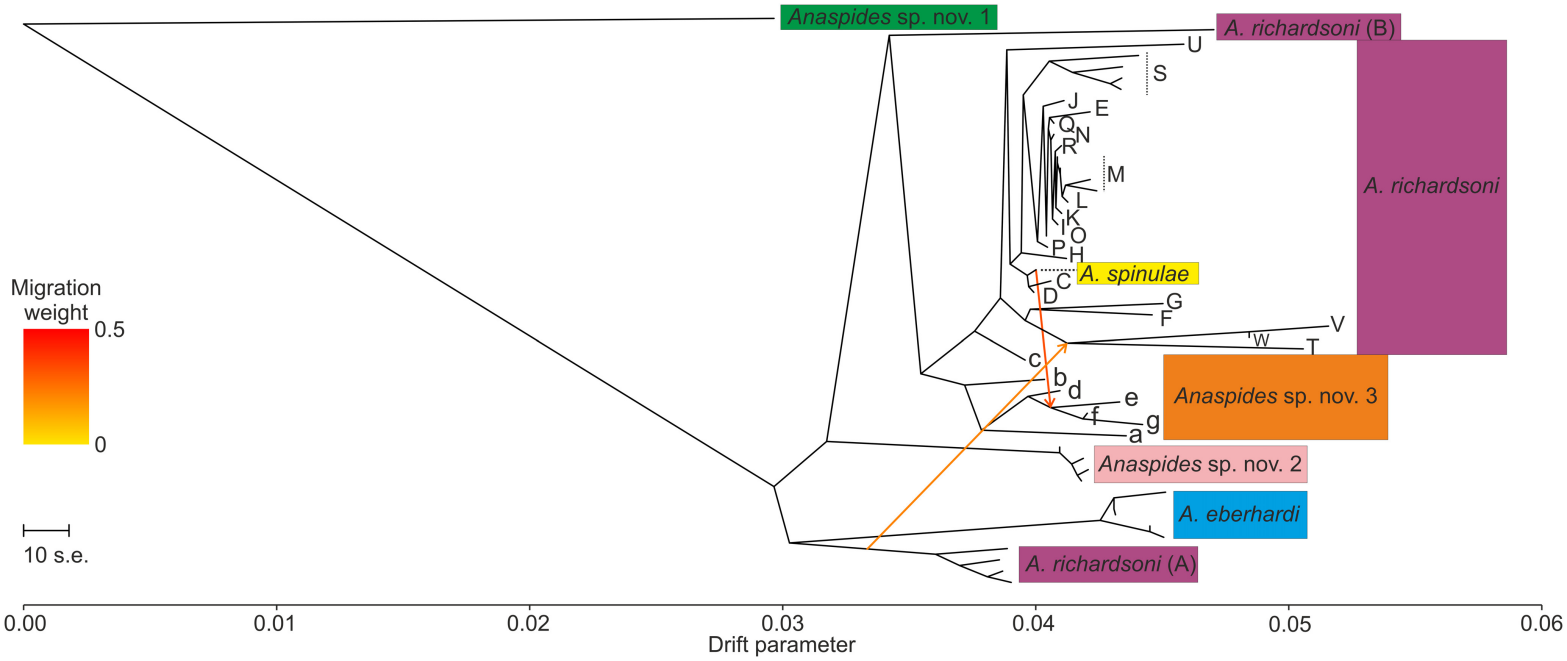
TreeMix analyses of data set 2 with *Anaspides* sp. nov. 1 set as outgroup (best parameter *m* = 2). Arrows correspond to possible gene flow events and colour to weight of the migration. Letters correspond to populations/metapopulations in Fig. 11. Coloured boxes on sides correspond to morphologically delineated species (based on Ahyong, 2015, 2016; Höpel et al., 2023a). Green: *Anaspides* sp. nov. 1, Purple: *A. richardsoni*, Blue: *A. eberhardi*, Light Pink: *Anaspides* sp. nov. 2, Orange: *Anaspides* sp. nov. 3, Yellow: *A. spinulae*. The phylogram was calculated with IQ‐Tree 2.3.6., Yellow: A. spinulae. The phylogram was calculated with IQ‐Tree 2.3.6.

Both the analysis of population structure with fineRADstructure and the phylogenetic analyses of the ddRAD data (see below, Figs 2 and 5) reveal a strong correlation between locality and lineages. Therefore, we assume that all conspecific specimens from a certain locality belong to the same population. For simplicity, we further combined (some) populations into metapopulations (Figs 2, 3, 4, 5, 6, 7, Figs S1 and S2, A, S and M).

Both phylogenetic analysis approaches (MP and ML) result in identical topologies with regard to the deep nodes and basal branching clades (*A. richardsoni* populations and metapopulations A, B, F, G, H, S, T, V, W, *A. eberhardi*, *Anaspides* sp. nov. 1, 2, 3) with high support values. All morphologically distinguished species, with the exception of *A. richardsoni*, represent discrete, monophyletic lineages. The same applies to phylogenetic networks reconstructed with SplitsTree. However, these analyses differ with respect to resolution and relationships among the more derived lineages (*A. richardsoni* C, D, E, I–R, U, *A. spinulae*). Especially in the MP consensus tree, these derived lineages were recovered as a polytomy. However, certain clades and lineages were recovered in both analyses. These clades were *A. richardsoni* C + D + *A. spinulae*, *A. richardsoni* N + E and several populations as discrete groups, *A. richardsoni*: U, I, J, O, P, Q, R. These results are also supported by the SplitsTree analyses (Fig. S1).

Our data show that *Anaspides* sp. nov. 1 is the most basal branching clade. Its genetically separate position is well supported by the PCA, STRUCTURE, fineRADstructure and SplitsTree analyses (Figs 2, 3, 4, 5, Fig. S1). This species is currently only known from a single lake on the eastern side of Mt. Field (Lake Nicholls) where it is syntopic with *A. richardsoni* A. Our data show no evidence of interbreeding between this species and *A. richardsoni* A, and no signs of shared genetic signal with any other herein studied species or population. This species can be readily separated from the remaining species and is sister group to what we herein refer to as the *A. richardsoni* species complex.

The major split of the *A. richardsoni* species complex shows *A. richardsoni* (A) and *A. eberhardi* from Mt. Field/Junee‐Florentine as the sister group to the remaining populations and species. The second clade consists of *Anaspides* sp. nov. 2 as the sister species to the remaining taxa. This species is found in and near cave waters in the Florentine Valley (Figs 1 and 11, pink‐coloured circles). The next lineage branching off is represented by an isolated population of *A. richardsoni* found in a cave at Lightning Plains (location B, exhibiting very slight cave adaptation, such as slight depigmentation, slight elongation of antenna, slight reduction of telson spines) followed by *Anaspides* sp. nov. 3. The latter species has been found in alpine tarns, lakes and caves to the south and south‐west of Lake St. Clair and the Franklin‐Gordon Wild Rivers National Park.

The remaining clade consists of several lineages of *A. richardsoni* as well as *A. spinulae*. Some of these lineages are further split into subgroups/sublineages mainly corresponding to certain localities that are important for understanding recolonisation of the central plateau (see below). With the exception of metapopulation M, each lineage represents a discrete locality (and also population, see Fig. 2). In the ML tree we distinguish 12 lineages of *A. richardsoni* (in the order: T/V/W, F/G, H, S, J, I/K/L/M/R, E/N, Q, O, P/U, C, D) that are gradually related to *A. spinulae*, with the basal branching clades (up to J) showing very high support values (>98) (Fig. 2). Note that the support values of all other nodes still exceed 59. The MP consensus tree is less resolved, but still recovered at least six lineages that are gradually related to *A. spinulae* (T/V/W, F/G, H, S, C, D) and several lineages (comprising the populations from E, I–R, U) with unresolved (polytomy) relationships with regard to *A. spinulae* + C + D. The bootstrap support values for the resolved lineages all exceed 65 (Fig. 2). The topology of the basal branching lineages in this clade is in both trees the same. The first lineage to branch off comprises populations found in the west and north‐west edge of the known distribution range, populations V (Lake Sandra), W (Tyndall Range) and T (Paddys Lake, Black Bluff Range) with the first two being sister groups (both West Coast Range). The following lineage shows populations F (Hydro Creek) and G (Mt Blackwood) as sister groups that are found on the eastern border of the range (eastern part of the Central Plateau). The next lineage comprises the specimens from population H (Liffey River) followed by the lineage comprising the populations of metapopulation S (Mole Creek).

The PCA (PC0‐1, PC2‐3) based on data set 2 of the *A. richardsoni* species complex reveals distinct genetic clusters for *A*. sp. nov. 1, *A*. sp. nov. 2, *A*. sp. nov. 3, *A. eberhardi* as well as *A. richardsoni* from Mt. Field (A), Lightning Plains (B), West Coast Range (W/V), whereas the remaining populations, all from the Central Plateau and Western Mountain Ranges (*A. richardsoni* C–U + *A. spinulae*) cluster together (Fig. 3I/II). Finally, data set 3 represents a subset of all samples that excludes *A*. sp. nov. 1, *A*. sp. nov. 2, *A. eberhardi* as well as *A. richardsoni* from Mt. Field (A) and is comprised of twice as many loci as the former data set. PCA analyses of this data set, which benefits from a higher genetic resolution for PCA due to the surplus of loci, show a clear‐cut separation of *A. richardsoni* populations/metapopulations C/D/*A. spinulae*, F/G, H, J, U, V, W (Fig. 3III/IV). Using data set 4, we can clearly separate *A. richardsoni* populations C, D and *A. spinulae* (Fig. 3V/VI), and in PC2, even the two morphological forms of *A. richardsoni* occurring in Clarence Lagoon (genetic lineage D, Figs 3 and 4).

We likewise carried out STRUCTURE analyses for data sets 2 and 3. For data set 2, 15 clusters were determined as best‐fitting to explain the genetic structure, while for data set 3, the best‐fitting *k*‐value was 11. Again *A*. sp. nov. 1, *A*. sp. nov. 2, *A. eberhardi* as well as *A. richardsoni* from Mt. Field (A) were uncovered as distinct clusters showing no sign of shared genetic coancestry (Fig. 4I). Also *A. richardsoni* from Lightning Plains (B) and *Anaspides* sp. nov. 3 form distinct clusters, but *A. richardsoni* from Lightning Plains (B) shows signals of minor genetic coancestry with *A*. sp. nov. 2 and *A*. sp. nov. 3. Moreover, *Anaspides* sp. nov. 3, especially population c, shares genetic ancestry with a group consisting of *A. richardsoni* populations C, D and *A. spinulae* (Fig. 4I/II). Regarding the *A. richardsoni* populations/metapopulations and *A. spinulae* from the Central Plateau/Western Mountain regions we can detect eight major groups having a distinct genetic signal. These groups are W/V, T, F/G, U, S, I–M/R, E/N–Q and the aforementioned clade comprising C/D/*A. spinulae*. However, we have detected shared genetic ancestry between some of those groups. The highest degree of shared genetic variation was found between I–M/R and E/N–Q. H is the only population that cannot be assigned to any of the other groups but seems to be composed of three shared ancestral components all at more or less similar ratios (F/G, E/N–Q, I–M/R). Moreover, the following groups also share some genetic signal: F/G + H, H + E–R, E/N–Q + H + S (one population), U + E/N–Q + C/D/*A. spinulae* (Fig. 4II).

In the fineRADstructure analysis of data set 2, we could detect 49 discrete genetic clusters corresponding largely to sampling localities (Fig. 5I, black boxes), with basically each sampling locality representing a separate cluster. The only exceptions to this are four sampling sites for *A. eberhardi*. The sampling sites “Nameless Spring” and “Settlement” (*F*_ST_ values 0.017, *d*_xy_ 0.0037, Figs S3 and S4) were recovered as one cluster, as also for the locations “Junee Cave” and “Gormenghast Cave” (*F*_ST_ value, 0.048, *d*_xy_ 0.0037, Figs S3 and S4). The sampling site “Clarence Lagoon” (genetic group D) shows two discrete clusters. Interestingly, these clusters correspond to two different morphological variations of *A. richardsoni* within the lagoon, with one exhibiting an overall higher degree of pleonal spination. However, as there were only five specimens examined that are most closely related to one another, have no other morphological differences and were found together at the same location, they are considered in the following as a single population.

The fineRADstructure analysis of data set 2 likewise supports the separation of the species, respectively lineages, of *A*. sp. nov. 1, *A*. sp. nov. 2, *A. eberhardi* as well as *A. richardsoni* from Mt. Field (A) and Lightning Plains (B). All are uncovered as discrete clusters with higher coancestry, and we did not detect any significant shared genetic variation between them. Both analyses (of data sets 2 and 3) also show both the separation of the populations of *Anaspides* sp. nov. 3 as well as of *A. richardsoni* (C–V) and *A. spinulae* from the Central Plateau (and adjacent mountain ranges; West Coast Range, Black Bluff Range, Cradle Mts). Moreover, particularly data set 3 allows us to distinguish groups that have a higher degree of shared coancestry such as *A. richardsoni* W/V, metapopulation S or *A. richardsoni* U/N. However, the shared coancestry between populations D, C and *A. spinulae* most likely represents common ancestry and not later introgression (particularly considering the phylogeny Fig. 2; also shown in the STRUCTURE analysis Fig. 4). Interestingly, there is also a signal of coancestry between *Anaspides* sp. nov. 3 population c and the group comprising *A. richardsoni* population C, D and *A. spinulae*, with the strongest signal to *A. richardsoni* population D. These populations all occur in close proximity. Furthermore, we detected slightly increased coancestry between the nearest populations to Lake St. Clair of *Anaspides* nov. sp. 3 (e, f and g) and *A. spinulae*.

The DSuite analyses uncover signals of historical gene flow, albeit most of these instances being found between populations of the same species. In *A. richardsoni* we can find strong signals of gene flow between some populations of metapopulation A, S and M as well as between populations P/O, N/L, H/G, L/M, T/F or J/P. In *Anaspides* sp. nov. 3, we could detect such gene flow between c and e/f/g. However, we can also find two instances of gene flow between species. One signal is between *Anaspides* sp. nov. 3 population c and the group/ancestral lineage comprising *A. richardsoni* C + D + *A. spinulae*. The second strong signal is between *A. spinulae* and the group comprising population e, f and g of *Anaspides* sp. nov. 3. While the first signal is found on both axes of the matrix, the second signal is only found on the *x*‐axis indicating probable introgression of *A. spinulae* into *Anaspides* sp. nov. 3 (Fig. 6, Fig. S2).

Using the OptM package, the best number of migration edges was *m* = 2. The first migration edge was found between the stem of *A. richardsoni* metapopulation A and the lineage comprising *A. richardsoni* populations T, V, and W. This gene flow event might indicate ancestral gene flow, respectively shared ancestry, between those two groups. The second migration event was detected between *A. spinulae* and the lineage comprising *Anaspides* sp. nov. 3 populations e, f and g and might indicate interbreeding between these two lineages, with introgression of *A. spinulae* into *Anaspides* sp. nov. 3 population e–g (Fig. 7).

### COI data

*Anaspides* sp. nov. 1 is not shown here, as it has a distinctly different cluster and 7.6–9.8% pairwise distance to all remaining species and populations examined in this study (all other species, respectively, populations have pairwise distances <7.3%).

**Fig. 8:**
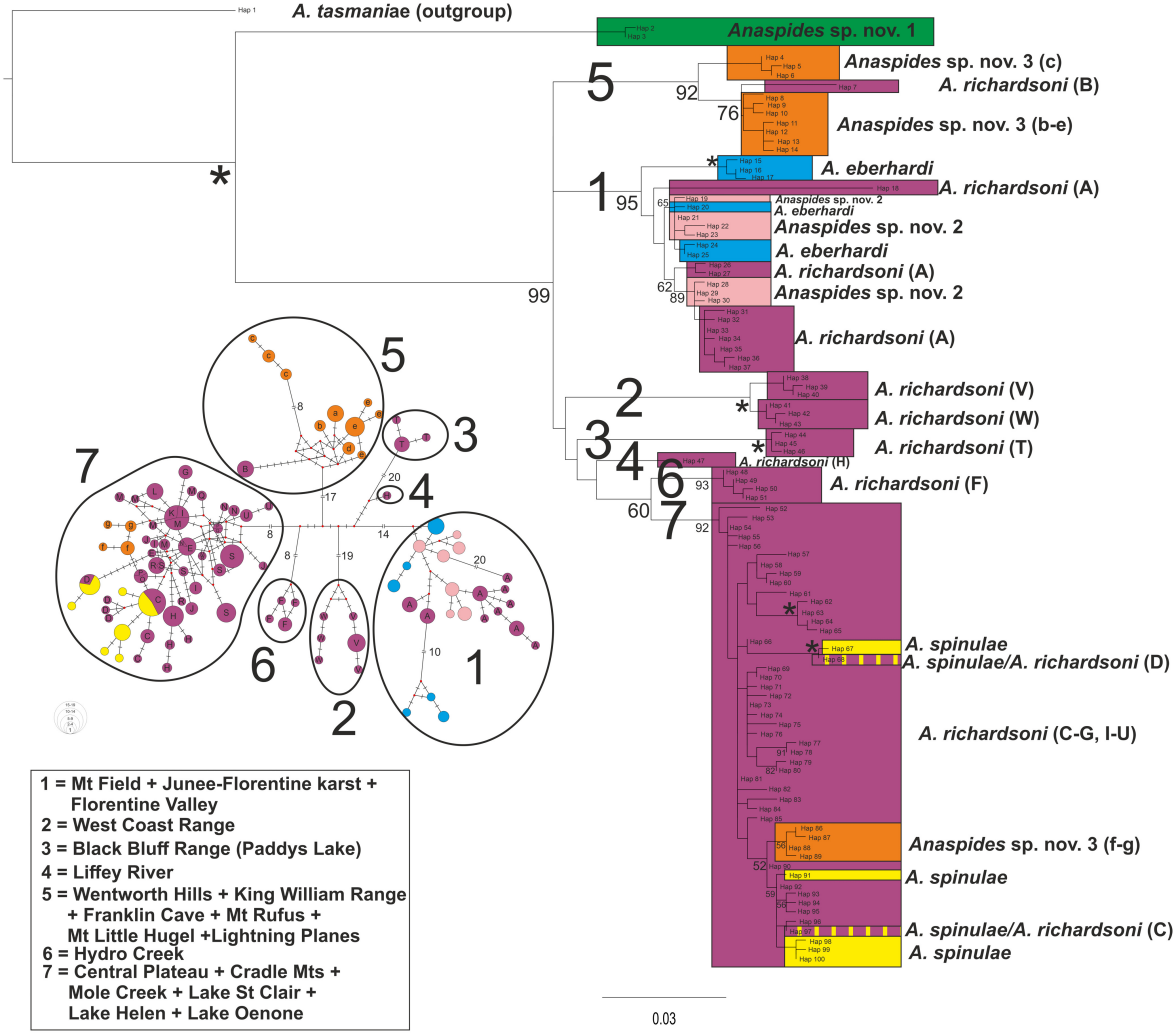
Maximum Likelihood phylogeny and median joining haplotype network of COI of the six species analysed in this study (323 individuals, 560 bp). The phylogeny was calculated with RaxML and *Anaspides tasmaniae* was used as an outgroup (HKY + G + I, 1000 iterations). The haplotype network was calculated with NETWORK 10.2.0.0 (Fluxus Technology Ltd.). Colours correspond to morphologically delineated species (based on Ahyong, 2015, 2016; Höpel et al., 2023a). Purple: *A. richardsoni*, Blue: *A. eberhardi*, Light Pink: *Anaspides* sp. nov. 2, Orange: *Anaspides* sp. nov. 3, Yellow: *A. spinulae*. Letters correspond to population/metapopulation in Fig. 11. Numbers correspond to major cluster (>3% pairwise distance).

In the COI haplotype network, seven major clusters (>3% pairwise distance) are present (Fig. 8). Cluster 1 comprises *A. richardsoni* from the Mt. Field area (metapopulation A), *A. eberhardi* and *Anaspides* sp. nov. 2, showing a complex structure with potential introgression, which will not be discussed in detail here. Cluster 2 comprises specimens from the West Coast Range (W/V), cluster 3 from the Black Bluff Range (T) and cluster 4 comprises one specimen from Liffey River (H). Population H (Liffey River) has also a second small cluster of haplotypes grouped within cluster 7. Cluster 5 comprises *Anaspides* sp. nov. 3 from populations a–e and *A. richardsoni*, population B. *Anaspides* sp. nov. 3 population c and *A. richardsoni* population B, however, show long branches within this cluster (>2% difference). Cluster 6 includes *A. richardsoni* from location F, and Cluster 7 comprises *A. richardsoni* from the Central Plateau and Cradle Mts (C–G, I–U), *A. spinulae*, and *Anaspides* sp. nov. 3 from populations f and g. Again, the presence of *Anaspides* sp. nov. 3 (f and g) in cluster 7 might indicate mitochondrial introgression into these two populations. Moreover, some populations/metapopulations have shared haplotypes such as the *A. richardsoni* populations I/K/M, E/N, M/R, O/P, P/Q, R/S as well as *A. spinulae*/*A. richardsoni* C and *A. spinulae*/*A. richardsoni* D. Moreover, *A. spinulae* together with *A. richardsoni* D have two distinct subclusters. The two subclusters in *A. richardsoni* D also correspond to the two morphological forms found in this habitat.

The phylogenetic analysis shows seven lineages corresponding to the seven clusters of the network (Fig. 8). At the base a polytomy between clusters 1, 5 and the rest (*A. richardsoni* C–W, *A. spinulae*, *Anaspides* sp. nov. 3 f–g) was recovered. The next off‐branching cluster is 2 (*A. richardsoni* V/W), followed by 3 (*A. richardsoni* T), 4 (*A. richardsoni* H), 6 (*A. richardsoni* F) and 7 (*A. richardsoni* C–G, I–U, *A. spinulae* and *Anaspides* sp. nov. 3 f–g). Within cluster 7, we find nested *A. spinulae* and *Anaspides* sp. nov. 3 (f–g). We can also distinguish one subcluster within cluster 7 comprising specimens belonging to *A. richardsoni* C, D, P, O, *A. spinulae* and *Anaspides* sp. nov. 3 (f–g). These areas (except O, P) are in close geographical proximity, belong to the same water system and were influenced by Pleistocene glaciation.

Because of the insufficient resolution in COI, paired with the multiple potential introgressions and nested position of some of the species, we do not apply an automatic species delimitation method for the COI data set.

## Discussion

The ddRAD data provide remarkable genetic resolution for most populations, where (almost) each sampling site represents an independent population (i.e. with higher genetic similarity of the individuals within the sampling site than with individuals from other sampling sites). There are few exceptions, and for the sake of simplicity, we summarized and collapsed some populations into metapopulations. The ddRAD data also indicate a limited dispersal potential of the individuals. This is particularly true for those specimens living in tarns and lakes but might be less strict for those individuals living in streams and creeks. The COI data are mostly concordant with the ddRAD data but provide less resolution, which might be due to substantially different evolutionary rates between mitochondrial and nuclear markers, incomplete lineage sorting and introgression. However, some of the lineages of the *A. richardsoni* species complex are well separated, indicating relatively old separation of the basal lineages. Both data sets also show more recent introgression of what is considered herein as separate species.

### Species delimitation and the case of *A. richardsoni* as a paraphyletic species

One of the currently undescribed species, herein called *Anaspides* sp. nov. 1, emerges as the sister species to all species of the *A. richardsoni* species complex (Fig. 2) in our analyses. *Anaspides* sp. nov. 1 is syntopic with *A. richardsoni* in Lake Nicholls (Mt. Field). Our genetic data (nuclear and COI) show no evidence of interbreeding between these two species, and they are morphologically distinct. *Anaspides* sp. nov. 1 clearly represents a separate species.

*Anaspides richardsoni*, as currently morphologically delineated (see diagnosis of Ahyong, 2016), represents a “paraphyletic” species relative to the remaining species in the species complex (*A. eberhardi*, *Anaspides* sp. nov. 2, *Anaspides* sp. nov. 3, *A. spinulae*) (Fig. 10). This formal description, however, does *not necessarily* represent biological reality but needs further consideration.

*Anaspides eberhardi*, *Anaspides* sp. nov. 2 and *Anaspides* sp. nov. 3 are well characterized by morphologically discrete characters, which, in our opinion, demonstrate ecological adaptations (*A. eberhardi*) and reproductive isolating mechanisms (*Anaspides* sp. nov. 2 and *Anaspides* sp. nov. 3). They are all monophyletic in the ddRAD data set. We consider the number of clasping spines as particularly important although we are aware that these differences alone might not result in a perfect (prezygotic) isolating mechanism. Differences also occur in the shape and length of the telson spines as well as the shape and setation of the inner antennular flagellum. We contend that the presence of only one clasping spine is the apomorphic condition for the *A. richardsoni* species complex, with three reversals to the plesiomorphic condition of two clasping spines, characterizing *Anaspides* sp. nov. 2, *Anaspides* sp. nov. 3 and *A. spinulae* (Fig. 10). The convergent reduction to one clasping spine in three *A. richardsoni* lineages (Mt. Field, Lightning Plains, Central Plateau/Western Mountain Ranges) would still require the reversal to two clasping spines in *A. spinulae*. We consider the convergent reduction to one clasping spine also as less plausible considering the high morphological similarity of all *A. richardsoni* lineages. In any case, *A. eberhardi*, *Anaspides* sp. nov. 2 and *Anaspides* sp. nov. 3 are “good” species according to the GLC.

**Fig. 9:**
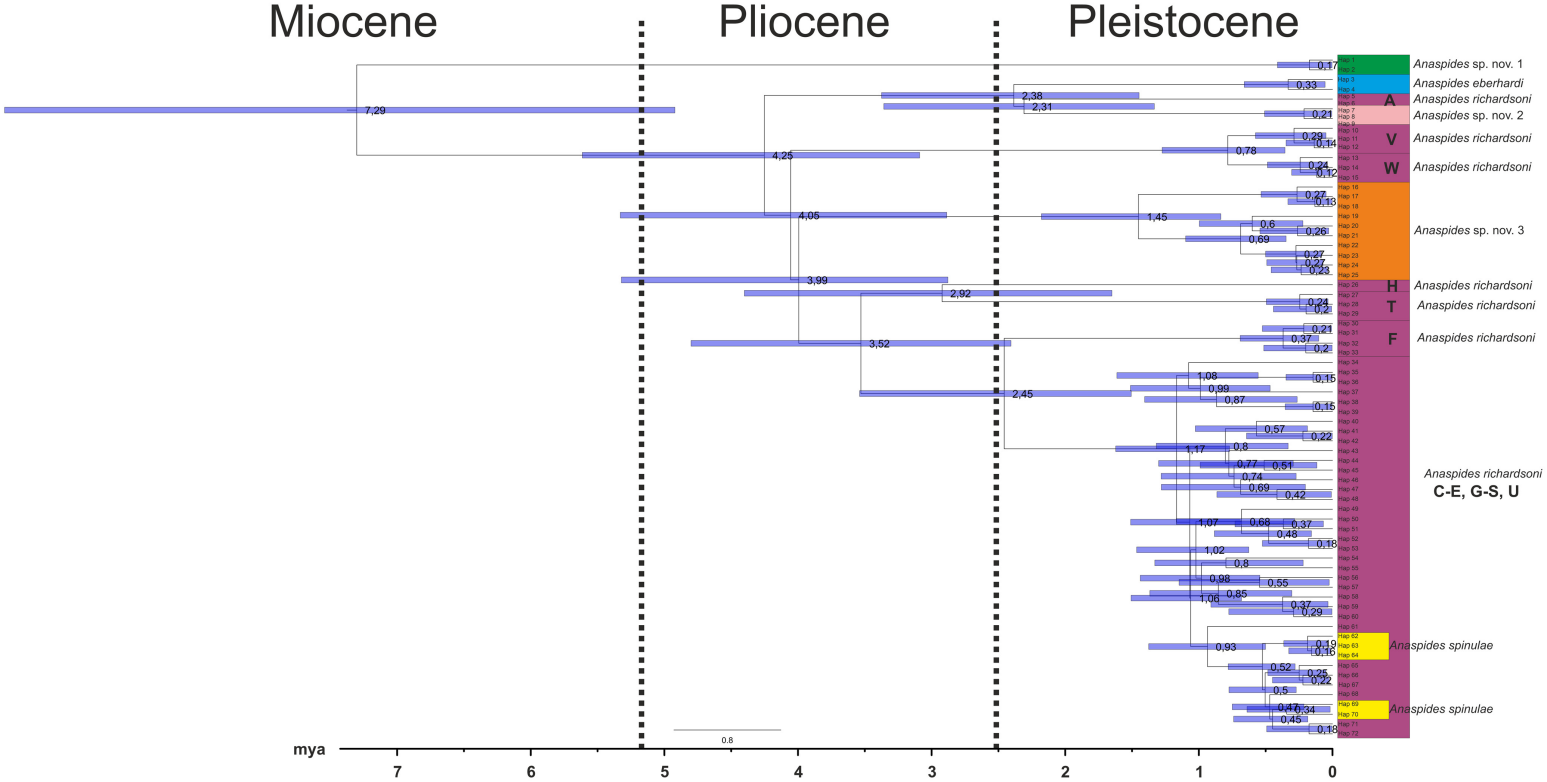
Strict molecular clock calculated with Beast V2 of COI of the six species analyses in this study (323 individuals, 560 bp). Letters correspond to populations/metapopulations in Fig. 11. We excluded lineages/haplotypes where we expect introgression and/or incomplete lineage sorting to have occurred (e.g. *Anaspides* sp. nov. 3 f‐g or *A. richardsoni* B). The split between the Mt. Field/Junee Florentine clade and the Central Plateau/Western Mountain Ranges clade has a median age of 4.25 Mya, and the split between the *Anaspides* sp. nov. 3 and those of the supposed refugial areas are only slightly younger (4.05 Mya) meaning that the same paleoclimatic event might have caused separation of the lineages.

For *A. richardsoni*, one could also argue for recognition to (at least) three “cryptic” species, “Mt. Field” (type locality, population A), “Lightning Plains” (population B) and “Central Plateau/Western Mountain Ranges/Mole Creek” (populations C to W), the latter potentially including *A. spinulae*. These (at least) three putative species are clearly allopatric, genetically distinct both in the ddRAD and the COI data sets. The split between “Mt. Field” and “Central Plateau” appears also to be relatively old (4.25 Mya, Fig. 9). Following this argument, each of the lineages (at least those six recognized in both phylogenetic analyses) grading towards *A. spinulae* might also be argued to represent separate species. For clarity, we deal with *A. spinulae* separately below. However, we hesitate to follow this approach for the following reasons. First, there are no diagnostic morphological differences between *A. richardsoni* populations A and C–W based on the careful and detailed study by Ahyong (2016), nor are there any known behavioural or ecological differences. The situation may be slightly different for population B from Lightning Plains because it shows slight troglomorphy. Only one of the “species criteria” from De Queiroz (1998, 2007) is fulfilled, which is being allopatric and reciprocally monophyletic. However, we cannot exclude the possibility of incipient speciation—not necessarily because of divergent ecological adaptation but long‐term genetic isolation. This may lead to post‐zygotic reproductive isolation owing to the emergence of Muller‐Dobzhansky incompatibilities in the form of incompatible novel mutations in the allopatric populations (Orr and Turelli, 2001).

**Fig. 10:**
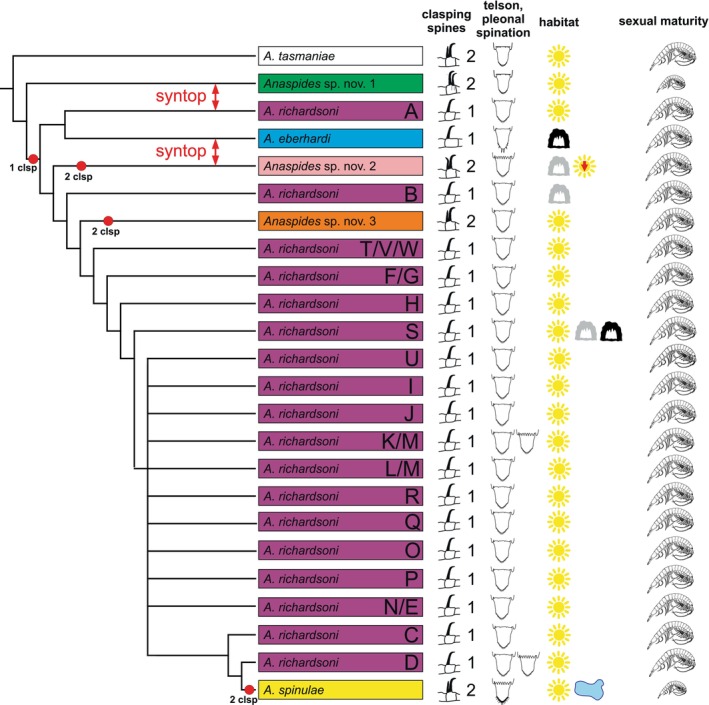
Scheme showing the relationships of the species of *Anaspides* which belong to the *Anaspides richardsoni* species complex with the two nearest outgroups (*A. tasmaniae* and *Anaspides* sp. nov. 1) and important morphological characters (number and position of clasping spines [clsp]., telson shape and spination of the telson and the tergal margins of pleonite 6, size of reaching sexual maturity) and habitat (sun = surface; red arrow = lowland >450 m, black cave = troglodytic, grey cave = cave inhabiting with slight cave adaptations, lake = big lake inhabiting).

The other more practical reason favouring treatment of *A. richardsoni* as a “paraphyletic” species for now is that separation of three or more species would lead to a great taxonomic and diagnostic instability—they are morphologically and ecologically indistinguishable. Recognition of three (or more) *subspecies* would be in the vein of Dufresnes et al. (2024) but still remain problematic because, although “inhabiting a geographic subdivision of the species”, they are not “differing taxonomically” (see Mayr, 1969). Notwithstanding that species delimitations represent hypotheses (Fitzhugh, 2009) and future analyses of more data might lead to different taxonomic conclusions, the recognition of new species within *A. richardsoni* should be done cautiously (Fig. 10).

The case of the *Anaspides* “central clade” is evolutionarily interesting as adaptive and non‐adaptive processes played crucial roles. On the one hand, the plesiomorphic morphology and absence of morphological differences, especially between the Mt. Field (A) and Central Plateau/Western Mountain Ranges (C–W) and among the C–W populations of *A. richardsoni*, are remarkable and seem to have prevailed over long evolutionary times (and potentially multiple speciation events). This suggests that this morphology is highly conserved and well adapted to the prevailing ecological conditions. This is sometimes referred to as “non‐adaptive radiation” (Gittenberger, 1991) but the question remains if this radiation necessarily leads to speciation. Our study also corresponds to the theory of allopatric speciation, where from several populations of originally one widespread species, some speciate (by sexual or ecological selection) and others do not (Mayr, 1942; Baker, 2005). On the other hand, several (good) species in our data set have evolved new morphological traits. In most cases, this involves a shift in the number of clasping spines that are assumed to play a role in mate recognition (Ahyong, 2015, 2016) and may thus contribute to prezygotic reproductive isolation. Such shifts can be observed in *A*. sp. nov. 2 and 3 and *A. spinulae*. They either occur syntopically with a single‐spined species (*A*. sp. nov. 2 and *A. eberhardi*) or show genetic evidence for past hybridization with such a single‐spined species (*A. spinulae* and *A. richardsoni* C/D; *A*. sp. nov. 3 and *A. richardsoni* C/D) and also often go hand in hand with a shift in habitats (surface to cave or lake). It seems plausible that in these cases, the secondary contact between young, recently diverging lineages leads to reinforcement through accelerated adaptive and segregating evolution (Noor, 1999). If the hybrids are less well adapted, the evolution of diverging mate recognition mechanisms (here the evolution of a second clasping spine) would increase the species fitness and reduce hybridization.

### *Anaspides spinulae* as a separate species

Probably, the most interesting case is that of *A. spinulae*. Our data indicate that several lineages of *A. richardsoni* from the Central Plateau/Western Mountain Ranges (Fig. 2) are successively related to *A. spinulae* in a grade. If considering *A. spinulae* a valid species, this again supports the paraphyly of *A. richardsoni* (as formally described). The minimum number of separate lineages gradually related to *A. spinulae* supported by our phylogenetic analyses is six (W/V/T, F/G, H, S, C, D, Fig. 2), depending on the analytical method. Even more striking is the very high genetic similarity between *A. spinulae* and *A. richardsoni* C + D (PCA, STRUCTURE, FineRADstructure, *F*_ST_ 0.025, *d*_xy_ 0.0039), also reflected by the shared haplotypes in COI, which suggest very recent speciation and rapid evolution of the morphological differences between these species. As with most *A. richardsoni* lineages, *A. spinulae* is recovered as a discrete (monophyletic) lineage in the tree and a discrete population in fineRADstructure. However, in contrast to the discrete *A. richardsoni* lineages/populations, *A. spinulae* shows discrete morphological and ecological adaptations, that is, the presence of two clasping spines, the strong, uneven spination of the telson and earlier onset of sexual maturity, which also speaks against the “ecomorph hypothesis” discussed by Williams (1965) when formally describing *A. spinulae*. We do not think that morphological plasticity and the presence of fish predators (e.g. galaxiids), such as seen in *Daphnia* (Adler and Harvell, 1990), played a major role in acquiring pronounced spination in *A. spinulae* and some populations of *A. richardsoni* (e.g. some specimens from population D and metapopulation M). Spiny and non‐spiny *A. richardsoni* sometimes occur syntopically (Clarence Lagoon), populations with pronounced spination occur in fishless waters (e.g. some locations in the Walls of Jerusalem, pool near Little Throne) and non‐spiny *A. richardsoni* occur syntopically with galaxiid fish (e.g. Lake Nicholls, Little Pine Lagoon, Pine Lake). Also, other species of *Anaspides* have not acquired pronounced spination although inhabiting lakes and occurring syntopically with fish (e.g. Lake Skinner, Weld River). As Ahyong (2016) noted, the distribution of the “spinulae” form of *A. richardsoni* correlates more strongly with geography than ecology and might reflect phylogeographic genotypic variation.

However, hybridization has been observed between *A. spinulae* (or more precisely the *A. spinulae*/*A. richardsoni* C/D group) and *A*. sp. nov. 3. There is evidence for two introgression events between *Anaspides* sp. nov. 3 and *A. spinulae* (or the common ancestor of *A. spinulae* and *A. richardsoni* C + D). One is between *Anaspides* sp. nov. 3 (population c; Wentworth Hills) and the actual ancestral populations C, D and *A. spinulae* (see STRUCTURE analyses; Treemix; Dsuite), which is not reflected in the COI data. However, in the analyses of COI data, we found a haplotype cluster of *Anaspides* sp. nov. 3 from locations f and g (Lake Helen, Lake Oenone; the geographically closest populations to Lake St Clair), which is only four mutations different from the cluster comprising *A. spinulae* and lineage C of *A. richardsoni*, suggesting potential mitochondrial introgression into these populations of *Anaspides* sp. nov. 3. These hybridization events may have transgressed the phenotype of two clasping spines from *Anaspides* sp. nov. 3 into *A. spinulae*, which would have been reinforced by segregation (Rieseberg et al., 1999) due to secondary contact with *A. richardsoni*. This raises again the question of whether the pronounced spination of the tergal and pleural margins of the pleonites, and especially of the telson, in combination with the smaller size at sexual maturity might be related to the lacustrine habitat of Lake St. Clair. Alternatively, a convergent reversal to 2 clasping spines in *A. spinulae* could have facilitated the introgression. In any case, we consider the apparent introgressions as not contradicting the presence of separate species, only that the complete reproductive isolation criterion might not yet have been completely fulfilled. In summary, we recognize *A. spinulae* as a very young species.

Potentially, the two genetically slightly diverging and morphologically differing syntopic lineages of *A. richardson*i D are at an early stage of adaptive and segregating evolution where the reproductive isolating mechanisms (i.e. diverging number of clasping spines) have not yet been established.

In summary, our multi‐gene analysis with well‐separated, allopatric populations fulfils the criteria of Coyne and Orr (2004) implying that “*A. richardsoni*” might indeed represent a “paraphyletic” species, whether or not populations A and B are considered (we are aware that the name *A. richardsoni* is connected to population A, from which the holotype comes). Paraphyletic species are also recognized among freshwater fish (Barluenga et al., 2006; Secci‐Petretto et al., 2023) and ground beetles (Fujisawa et al., 2019). Fujisawa et al. (2019) uncovered paraphyletic relationships for *Carabus maiyasanus* and *C. iwakanianus*, as well as *C. arrowianus* being paraphyletic in regard to three other species (all having parapatric boundaries with *C. arrowianus* being the central species sharing boundaries with all other species). Paraphyly also occurs within the *S. salamandra* species clade, which comprises over 12–17 subspecies, one sometimes being considered a separate species (mostly based on coloration patterns and geographic distribution). Burgon et al. (2021) suggest at least four instances of paraphyletic subspecies. Secci‐Petretto et al. (2023) show that the grayling *Thymallus baicalensis* (widespread species in Siberia and Mongolia, mainly found in rivers and near outlets of lakes including the outlet of Lake Hovsgol) is paraphyletic with respect to *T. nigrescens* (only found in Lake Hovsgol, with different ecological adaptations). Roman et al. (2018) suggest a very recent divergence of *T. nigrescens* (32 kyr). This example is strikingly similar to the case of *A. spinulae*.

### Paleoecological reconstruction of extant distribution of Central Plateau/Western Mountain Ranges *Anaspides*

The influence of the Pleistocene glacials and interglacials on the distribution of animals and plants has been long discussed (e.g. de Lattin, 1967). In particular, higher elevations were covered with ice shields but also non‐glaciated areas were influenced by changing climatic conditions. Individuals of mobile species (e.g. birds, many insects) might have dispersed to the refugial areas, whereas less mobile (e.g. soil animals, but probably also certain limnic taxa) might have become extinct in these areas. In the interglacials and after the LGM, these areas have become recolonized from so‐called glacial refugia. Crustaceans are not well studied in terms of Pleistocene paleoclimate changes (e.g. Puli et al., 2024) and the “central clade” *Anaspides* might be an excellent system for such study.

Since the Early Miocene, Tasmania was carried north due to plate tectonics on the Australian Plate from 65° latitude and reaching its present position at 42° latitude around the Late Miocene (Gallagher et al., 2003; Corbett, 2019). Around this time, the Antarctic started to freeze over with the biggest fluctuation of glacial extent during the Pliocene (Warnke et al., 1992). This also led to variations in the relative dominance of rainforest and wet sclerophyll forests in south‐eastern Australia through that time period (Macphail, 1997). Gallagher et al. (2003) showed that in the early Pliocene the climate was warm and wet with a peak in summer rainfall (mean annual temperature 2–4 °C higher than present, mean annual precipitation 50–70% higher than present), whereas the climate at the end of the Late Pliocene was drier and cooler with a winter rainfall peak (mean annual temperature 0–2 °C higher than present, mean annual precipitation 0–30% higher than present) We thus assume that *Anaspides* (specifically the *A. richardsoni* complex) inhabited a continuous range in Central Tasmania including Mt. Field, the Central Plateau, the Western Mountain Ranges and adjacent landscapes already during the Pliocene.

**Fig. 11:**
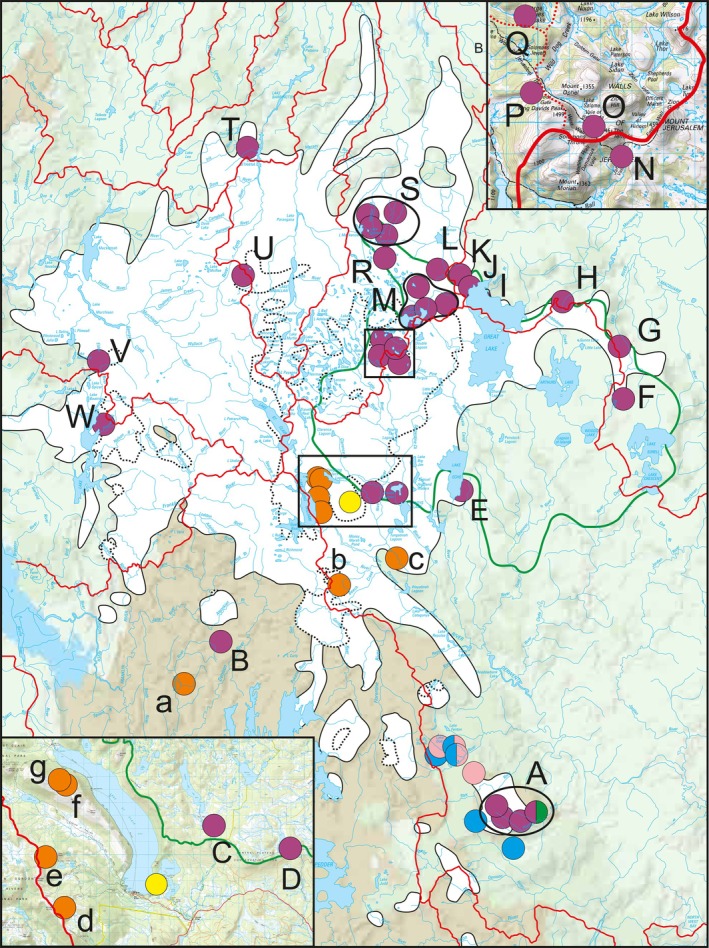
Distribution map of the six species of *Anaspides* examined in this study with watersheds (red lines) and maximum glaciated area of the Central Plateau (green line) during the Linda Glaciations (closed black lines, white) after Corbett (2019); Houshold and Jackson (2023) and during the Last Glacial Maximum (LGM, 22 000–17 000 Mya) (dotted black lines, white) after Colhoun et al. (1996). The Central Plateau is marked with a green outline (after Corbett, 2019). Purple: *A. richardsoni*; Yellow: *A. spinulae*; Blue: *A. eberhardi*; Green: *Anaspides* sp. nov. 1 from Lake Nicholls; Pink: *Anaspides* sp. nov. 2 from the Florentine Valley; Orange: *Anaspides* sp. nov. 3. Half‐circles indicate syntopic.

In the following, we will focus on the *A. richardsoni* populations from the Central Plateau/Western Mountain Ranges C–W as well as *A. spinulae*. Lineages T, U, V, and W belong to the Western Mountain Regions, while lineages C–S lie directly on or on the edges of the Central Plateau (Fig. 11, green outline). The locations on the edges are E, I, J, K, which are creeks draining from the Central Plateau (specimens in region K are found on the Plateau as well as on the slopes) as well as the caves which are found in region S.

We propose that Cenozoic glaciations (the latest being the so called Linda glaciations in the early Pleistocene; Colhoun et al., 2010) may have wiped out most of the populations on the western and northern part of Central Plateau and Western Mountain Ranges and left or forced *Anaspides* to regions at and/or beyond the ice margins. Subsequent glaciations are often referred to as being in general less extensive, giving the potential that habitats uninhabitable during previous glaciations may have served as new and additional refugia and subsequently as stepping stones and sources for recolonization, which may have been continuously inhabited throughout even during later glaciation events. It is likely that some of the diverging genetic lineages of *A. richardsoni* evolved and persisted in these refugial areas, though we do not know their exact localities. Other regions may have been (re‐)colonized once or repeatedly from one or multiple refugia; the genetic differentiation of these recolonized populations is expected to be less pronounced than in the continuously inhabited regions and the chance for hybridization from different long‐term refugia increases. In any case, the extant distribution at higher elevations is the result of a post‐glacial recolonization. Lowland areas, which are not inhabited by *Anaspides* today, might have been suitable habitats during that time, as temperatures were 6–10 °C cooler than the present and the snowline was lower by 1000–1500 m (Colhoun et al., 2010); also the sea level was lowered by 130 m during the LGM (Morrison et al., 2023). An exception is *Anaspides* sp. nov. 2, which still occurs in lowland creeks (>450 m) near the outflow of caves, which supply cold and clear water.

With regard to the genetic clusters uncovered in the STRUCTURE analyses (k11), we have to assume several glacial refugia during the Pleistocene (or earlier) glaciations regarding *A. richardsoni* populations from the Central Plateau/Western Mountain Ranges C–W as well as *A. spinulae*. The exact area of the glacial refugia can only be determined vaguely. However, it is assumed to be at least in proximity to the ice margins and present‐day areas, in adjacent landscapes, lowland areas and in some cases potentially even the same waters (e.g. F). These glacial refugia are mainly represented by the lineages comprising the basal branching populations and two groups containing the majority of populations in the central and western part of the Central Plateau. For the basal branching lineages, we assume glacial refugia to the west (lineage W/V), north‐west (lineage T) and east (lineage F/G) of the glaciated area. Our molecular clock analysis dates the separation of the basal branching lineages between 2.45 and 4.05 Mya, which is slightly older than early Pleistocene glaciations (Linda glaciations). This could either be a miscalibration of the molecular clock itself (no fossils or geological events were used for calibrating) or could indicate that these populations were already separated before the Linda glaciations. This could have been caused by a potential earlier glaciation during the Pliocene (Macphail et al., 1993; Augustinus and Idnurm, 1993). The Pleistocene glaciations then only reinforced a further separation of these populations. In any case, those populations geographically in‐between the separated ones became entirely extinct.

The discrete cluster in the STRUCTURE analyses also indicates that the caves inhabited by metapopulation S could have acted as a separate refugium, which will be in the focus of another study. For group C–E/I–R, the potential refugia could have been on the north‐eastern (I–R) and south‐eastern (C, D, *A. spinulae*) parts of the glaciation. The north‐eastern refugium could have been situated near the areas of today's populations H–K. Our molecular clock also dates the first split of the clade comprising C–E/I–S to 1.17 Mya, which is substantially younger than the split of the basal branching lineages. This leads us to believe that the glacial refugia for the populations U, *A. richardsoni* C/D, and *A. spinulae* became only glacial refugia during the subsequent (mid‐Pleistocene) glaciations. This is also in concordance with the heavy glaciation of these areas and adjacent landscapes during the Linda glaciations.

Our data give evidence that the main recolonization of the western and central parts of the Central Plateau (I–R) happened from the refugial area inhabited by the ancestors of H and, to a minor part, also from the refugium of *A. richardsoni* C/D and *A. spinulae*, seen by the shared genetic information in the STRUCTURE analyses (k11 light and dark blue colours). As the populations N–Q (Walls of Jerusalem) also exhibit a certain genetic cluster comprising two different genetic components, they could represent a separate glacial refugium during one of the later glaciation events, as the area was ice free during the LGM. Interestingly, population E, although located at the ice margins (south‐east border), probably never acted as a refugial population because of the derived position in the tree and the close genetic proximity (sister groups in ddRAD, shared haplotype in COI) to population N in the northern part of the Plateau. This area must have been recolonized from the northern part of the Central Plateau (Walls of Jerusalem, populations N, O, P, Q), which is one of the only instances so far of “long distance” dispersal (for a small mountain shrimp) in *Anaspides*. This migration can be well explained through the fact that populations N and E belong to the same water system, even to the same river system (Pine River) with N being the headwaters region.

Despite substantial glaciation during the LGM (20000–12000 years ago) only a few of the herein studied populations and areas would have been directly affected by those glaciers (Fig. 11). These areas comprise the populations in and around the margins of Lake St Clair (*Anaspides* sp. nov. 3 d–g, *A. spinulae* and *A. richardsoni* C–D). Given that Lake St. Clair was glaciated during the LGM, this might indicate that *A. spinulae* would have evolved after that time. This is in contrast with the earlier views of Williams (1965) that *A. spinulae* survived the Pleistocene glaciation of Lake St. Clair in adjacent periglacial lakes or melt‐waters and also with the hypothesized in situ refugium in deeper parts of the lake proposed by Ahyong (2016). Lake St Clair offers a very different environment than the substantially smaller mountain tarns, pools and runnels otherwise inhabited by *A. richardsoni*. Thus, after colonization of Lake St. Clair, probably from the refugial region of *A. richardsoni* C/D, the lake population quickly adapted to the new lacustrine environment. At that time, *A. richardsoni* may have dispersed more frequently to Lake St. Clair, favouring segregating mate recognition mechanisms. In fact, this may be a case of character displacement (Brown and Wilson, 1956). It might therefore represent an interesting case of parapatric or even sympatric speciation (Coyne and Orr, 2004; Barraclough, 2019) which needs further consideration. Such cases of speciation with gene flow are also known from *Heliconius* butterflies (Martin et al., 2013), *Myotis* bats (Morales et al., 2017) and ground beetles (Fujisawa et al., 2019). Moreover, Fujisawa et al. (2019) state that the genomic divergence pattern among *Carabus* (*Ohomopterus*) beetles demonstrates how divergence of a key trait for reproductive isolation (genital morphology) may result in increased species richness despite occasional gene flow due to hybridization.

Based on the COI molecular clock, it appears also reasonable to suggest that the same paleoclimatic event that was responsible for the separation of the basal branching lineages of the Central Plateau/Western Mountain Ranges (2.45–4.05 Mya) was also responsible for the earlier separation (4.25 Mya) between the Mt. Field/Junee‐Florentine species group and the Central Plateau/Western Mountain Ranges species group. The glaciations certainly blocked the connection between north and south in what are today the Gordon and the Derwent Rivers. This event would also include the separation of *Anaspides* sp. nov. 3 from the other lineages. Given the deep nodes and clusters in the STRUCTURE analyses, *Anaspides* sp. nov. 3 (or its *A. richardsoni*‐like progenitor) might have had its glacial refugium during the Linda glaciations in the south‐eastern part of its distribution, close to the areas of population c (Wentworth Hills) and b (King William Range). From here it must have recolonized the areas in the north west of Lake St Clair comprising the populations d–g (all branching off in that order, which is also the specific geographical order from south to north).

## Conclusion

Our data show a remarkable resolution on the population level with essentially each water body having its own distinct *Anaspides* population. This underlines the low dispersal ability of *Anaspides* and the high potential for allopatric speciation, especially in combination with different geomorphological processes, such as glaciation. We show that even these glaciations played a major role in shaping the current distribution of *Anaspides richardsoni* and *Anaspides spinulae* populations, with several glacial refugial areas. Most of these refugial populations might not have migrated very far (if at all), while the final recolonization of the western and central parts of the Central Plateau derived from two refugia to the north‐east and south‐east of the plateau, respectively. However, the question remains: do genetically distinct populations represent species or not? This has significant conservation implications. It is not only necessary to protect individual species but also to protect the many separate populations (which might represent incipient species) and areas where they exist.

## Conflict of interest

None declared.

## Supporting information


**Fig. S1.** Unrooted phylogenetic network of ddRAD data set 1 (261 individuals, 1945 polymorphic loci with 38 926 variable sites, TVM + G + I, 1000 iterations) calculated with SplitsTree4 v4.19.2 (Huson & Bryant, 2006) with the Neighbor‐Net algorithm and uncorrected *p*‐distances.

**Fig. S2.** DSuite analysis of data set 3 with *Anaspides richardsoni* population B set as outgroup.

**Fig. S3.** *F*_ST_ values using data set 2.

**Fig. S4.** *d*_xy_ values using data set 2, (I) with *Anaspides* sp. nov. 1 and (II) without *Anaspides* sp. nov. 1.

**Table S1.** List of individuals used for this study.

**Table S2.** *F*_ST_ values using data set 2.

**Table S3.** *d*_xy_ values using data set 2.

## Data Availability

The data that support the findings of this study are openly available in https://github.com/ch515/Hoepel_paraphyletic_species. All sequences and genetic data are uploaded to NCBI. COI: PV780789–PV781115; ddRAD: SAMN49064136–SAMN49064398, BioProject ID: PRJNA1276447.
