# Supplementary figures and images for: Phylogeography of the Anaspides richardsoni species clade (Anaspidacea, Anaspidesidae): glaciation and recolonization of the Tasmanian Central Plateau and the question of paraphyletic species
Source: Cladistics. 2025 Jul 28;41(5):470–92. doi: 10.1111/cla.70005 (PMC12466092; doi:10.1111/cla.70005)

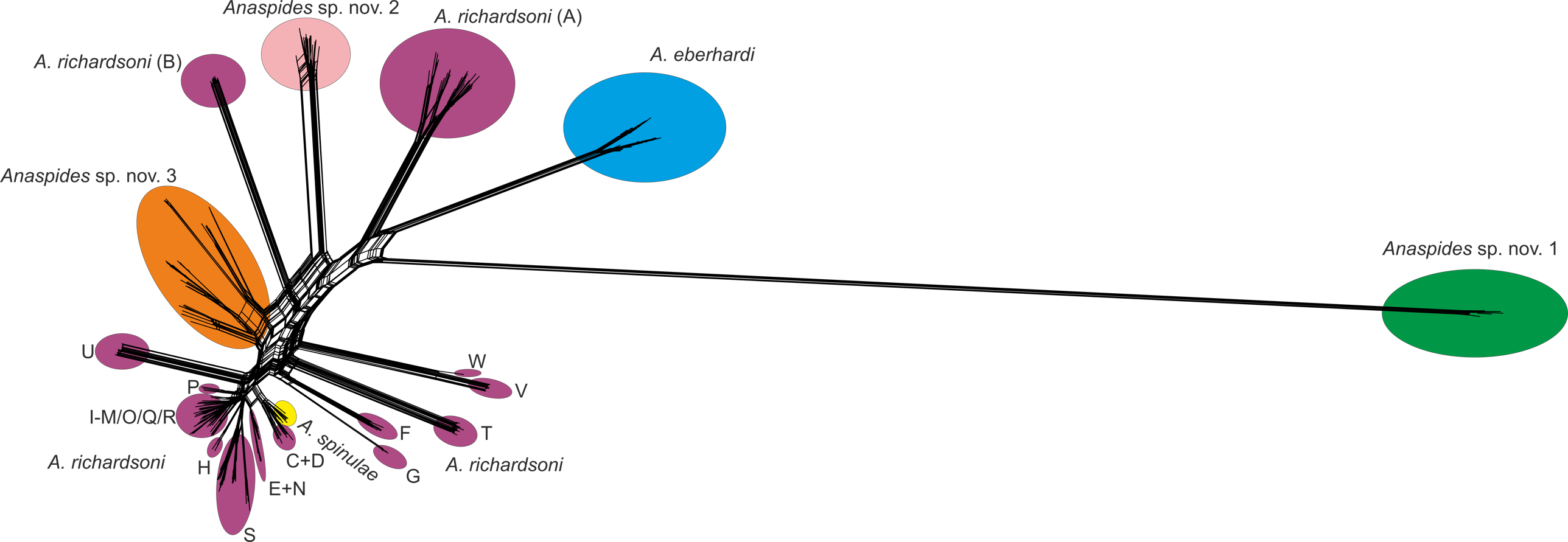

Supplement: Supplementary file 1 — Fig. S1. Unrooted phylogenetic network of ddRAD data set 1 (261 individuals, 1945 polymorphic loci with 38 926 variable sites, TVM + G + I, 1000 iterations) calculated with SplitsTree4 v4.19.2 (Huson & Bryant, 2006) with the Neighbor‐Net algorithm and uncorrected p‐distances. [file CLA-41-470-s007.png]

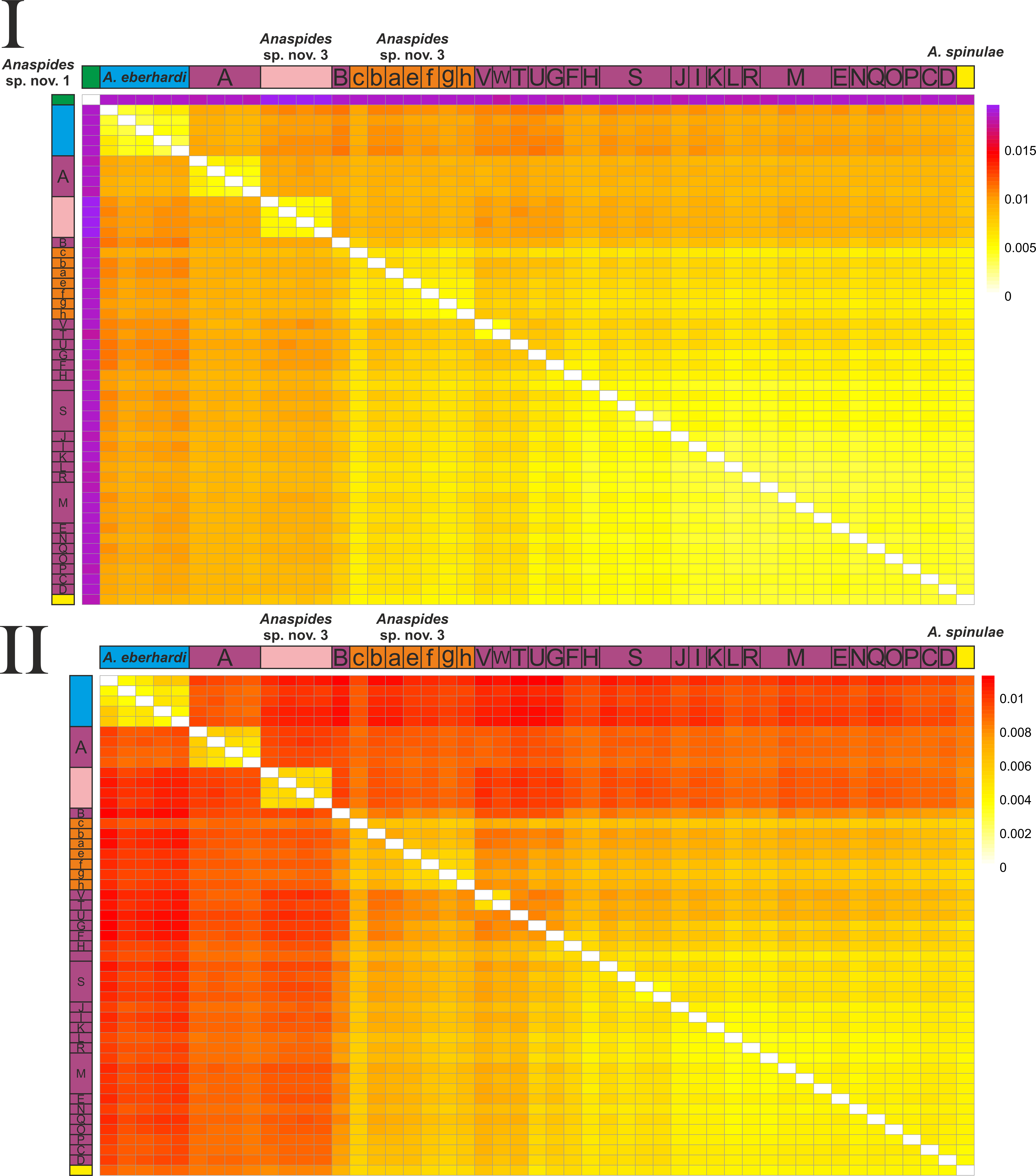

Supplement: Supplementary file 4 — Fig. S4. d xy values using data set 2, (I) with Anaspides sp. nov. 1 and (II) without Anaspides sp. nov. 1. [file CLA-41-470-s004.png]
